# Improving measurement of child abuse and neglect: A systematic review and analysis of national prevalence studies

**DOI:** 10.1371/journal.pone.0227884

**Published:** 2020-01-28

**Authors:** Ben Mathews, Rosana Pacella, Michael P. Dunne, Marko Simunovic, Cicely Marston

**Affiliations:** 1 Director, Childhood Adversity Research Program, Faculty of Health, Queensland University of Technology, Brisbane, Queensland, Australia; 2 Adjunct Professor, Johns Hopkins University, Bloomberg School of Public Health, Baltimore MD, United States of America; 3 Institute for Lifecourse Development, University of Greenwich, Greenwich, London, United Kingdom; 4 School of Public Health, Faculty of Health, Queensland University of Technology, Brisbane, Queensland, Australia; 5 Institute for Health and Biomedical Innovation, Queensland University of Technology, Brisbane, Queensland, Australia; 6 Faculty of Public Health and Policy, London School of Hygiene and Tropical Medicine, London, United Kingdom; University of South Florida, UNITED STATES

## Abstract

**Objectives:**

Child maltreatment through physical abuse, sexual abuse, emotional abuse, neglect, and exposure to domestic violence, causes substantial adverse health, educational and behavioural consequences through the lifespan. The generation of reliable data on the prevalence and characteristics of child maltreatment in nationwide populations is essential to plan and evaluate public health interventions to reduce maltreatment. Measurement of child maltreatment must overcome numerous methodological challenges. Little is known to date about the extent, nature and methodological quality of these national studies. This study aimed to systematically review the most comprehensive national studies of the prevalence of child maltreatment, and critically appraise their methodologies to help inform the design of future studies.

**Methods:**

Guided by PRISMA and following a published protocol, we searched 22 databases from inception to 31 May 2019 to identify nationwide studies of the prevalence of either all five or at least four forms of child maltreatment. We conducted a formal quality assessment and critical analysis of study design.

**Results:**

This review identified 30 national prevalence studies of all five or at least four forms of child maltreatment, in 22 countries. While sound approaches are available for different settings, methodologies varied widely in nature and robustness. Some instruments are more reliable and obtain more detailed and useful information about the characteristics of the maltreatment, including its nature, frequency, and the relationship between the child and the person who inflicted the maltreatment. Almost all studies had limitations, especially in the level of detail captured about maltreatment, and the adequacy of constructs of maltreatment types.

**Conclusions:**

Countries must invest in rigorous national studies of the prevalence of child maltreatment. Studies should use a sound instrument containing appropriate maltreatment constructs, and obtain nuanced information about its nature.

## Introduction

Child maltreatment is common and causes substantial adverse health, educational and behavioural consequences [1]. Understanding its prevalence and characteristics in nationwide populations is essential to plan and evaluate interventions to reduce maltreatment. However, measurement of child maltreatment is known to be far from universal, and when performed must confront methodological challenges. This study systematically reviews the most comprehensive national studies of the prevalence of child maltreatment, and critically appraises their methodologies to help inform future measurement.

Child maltreatment in its five recognised forms is a major public health issue [2]. Physical and mental diseases are caused through proximal and distal pathways. Immediate physical injuries and conditions include brain injury and failure to thrive, and a panoply of psychological disorders include anxiety, depression, and suicidality. Studies have found serious effects of physical abuse [3,4,5], sexual abuse [6,7], emotional abuse [5,8–10], neglect [5,9,11], and exposure to domestic violence [12–14]. Experiencing multiple forms of maltreatment is common [12], and is associated with more severe outcomes [14,15], including alcohol and drug abuse, mental illness, interpersonal violence, and sexual risk taking [16].

The adoption of coping mechanisms such as smoking, alcohol and drug use can compound the damage by causing diseases in the medium to long term; and chronic stress can cause coronary artery disease, pulmonary fibrosis, and inflammation [17–22]. Potent mediators include prolonged psychological distress, risky behaviours, social withdrawal and dysfunction, impaired cognitive development, low educational and occupational attainment, and interpersonal relationship difficulties. A growing body of evidence is showing child maltreatment affects brain development, shortens telomeres, and produces epigenetic neurobiological changes [23–26]. The disease and economic burdens are substantial: a recent estimate of the cost of disability-adjusted life years (DALYs) lost across East Asia and the Pacific was 1.88% of the region’s GDP, equating to $194 billion in 2012 dollars [27].

As a global policy imperative, the United Nations recognises the gravity of child maltreatment and its consequences. The United Nations Agenda for Sustainable Development includes a target of ending abuse of children [28]. Reliable scientific data on national prevalence is essential to measure progress against this goal, and to inform policy efforts aimed at prevention, early identification and response [29–30].

Nationwide studies of the experience of childhood maltreatment can identify baseline prevalence stratified by maltreatment type, as well as important contextual features including the child’s sex, age, and relationship with the abusive person. Without good measurement techniques and repeated measures over time, we lack understandings of baseline measures, of whether maltreatment is increasing or declining, of changes in maltreatment types over time, and of the efficacy of policy and practice interventions designed to reduce child maltreatment for the whole population and for key sub-populations.

Despite the necessity for good data in public health generally and in child maltreatment in particular, approximately half of all countries have failed to report any kind of robust prevalence estimates [2], and extant studies are often limited to measuring one or few maltreatment types [31]. Accordingly, prevalence estimates are often inadequately specified, and are almost certainly underestimated. In addition, existing studies vary widely in design, sample and methods, and often use non-standardized instruments [5,32]. Where an instrument is non-standardized and untested, the risk may be heightened that the study will fail to capture experiences that constitute maltreatment, and may capture experiences that do not constitute maltreatment, hence producing unreliable results. Importantly, the use of unsound maltreatment constructs and operational definitions also compromises the reliability of recorded measures [33–34]. As an example of this, studies of sexual abuse that do not include non-contact sexual abuse will underestimate prevalence; conversely, studies that include as sexual abuse genuinely consensual acts between peers will overestimate prevalence. Similarly, studies of neglect that do not consider medical neglect will underestimate prevalence. Studies of emotional abuse that include non-abusive yelling will overestimate prevalence.

Optimal methodologies for measuring population characteristics of child maltreatment can ensure adequate detail is captured to yield reliable, detailed, useful data. For best quality estimates, prevalence studies should adopt robust conceptual understandings of maltreatment types and their operational definitions [33]. In addition, prevalence studies need to ask a series of items to obtain accurate data, rather than a single question which will tend to underestimate prevalence [35]. Similarly, to avoid underestimates, items should be behaviourally specific, rather than vague, ambiguous or non-specific [36]. All national prevalence studies face methodological and practical challenges, and studies take different approaches [2,12,14,30]. Ideally, however, all five forms of child maltreatment should be measured simultaneously, since many children experience such poly-victimization and its heightened consequences [1,14,16]. To provide nuanced, useful information, studies should ask about prevalence, and about the specific nature of the acts, their severity, frequency, and timing, and the relationship of the child to the person inflicting the abuse [33]. These factors influence health outcomes and provide evidence about specific risk and protective factors and how these may best be targeted. Rigorous measurement of child maltreatment is complex, but is essential to inform prevention efforts and drive nationwide social change [2,14,29,36,37].

Recent research has reviewed global prevalence estimates [2,31], the nature of population health surveys exploring consequences of child maltreatment [37], and approaches in studies of youth [38]. However, to date, there has not been a systematic review and methodological appraisal of high quality national population prevalence studies of child maltreatment to provide a baseline for future measurement efforts.

This study aimed to investigate three questions. First, what national studies have been conducted of the prevalence and nature of all five, or at least four, major forms of child maltreatment? Second, what methodologies were used in these studies? Third, what does a critical analysis of these studies indicate about the methodological rigour, quality, and practical viability of different approaches? The results of our investigation can inform future efforts to generate baseline prevalence estimates, design policy responses, and chart trends over time, as more societies confront the challenge of childhood maltreatment.

## Methods

### Search strategy

Our systematic review was guided by PRISMA [39] (S1 Fig). We developed a protocol, registered at PROSPERO [40]. #CRD42017068120, https://www.crd.york.ac.uk/PROSPERO). Adopting our search strategy (S1 File), we searched 22 databases from their inception to 31 May 2019.

### Eligibility criteria

We searched for quantitative studies of the prevalence of child maltreatment. Included studies met four criteria: (1) primary empirical studies of the prevalence of four or five types of child maltreatment: ((i) physical abuse; (ii) emotional or psychological abuse; (iii) neglect; (iv) exposure to domestic violence; and (v) sexual abuse; (2) studies conducted nationwide using a representative sample of the population; (3) studies involving adult or child participants providing self-reported information about their experience, or studies where adults provided information about their child’s experience; (4) peer-reviewed studies or substantial grey literature.

### Screening

**Fig 1:**
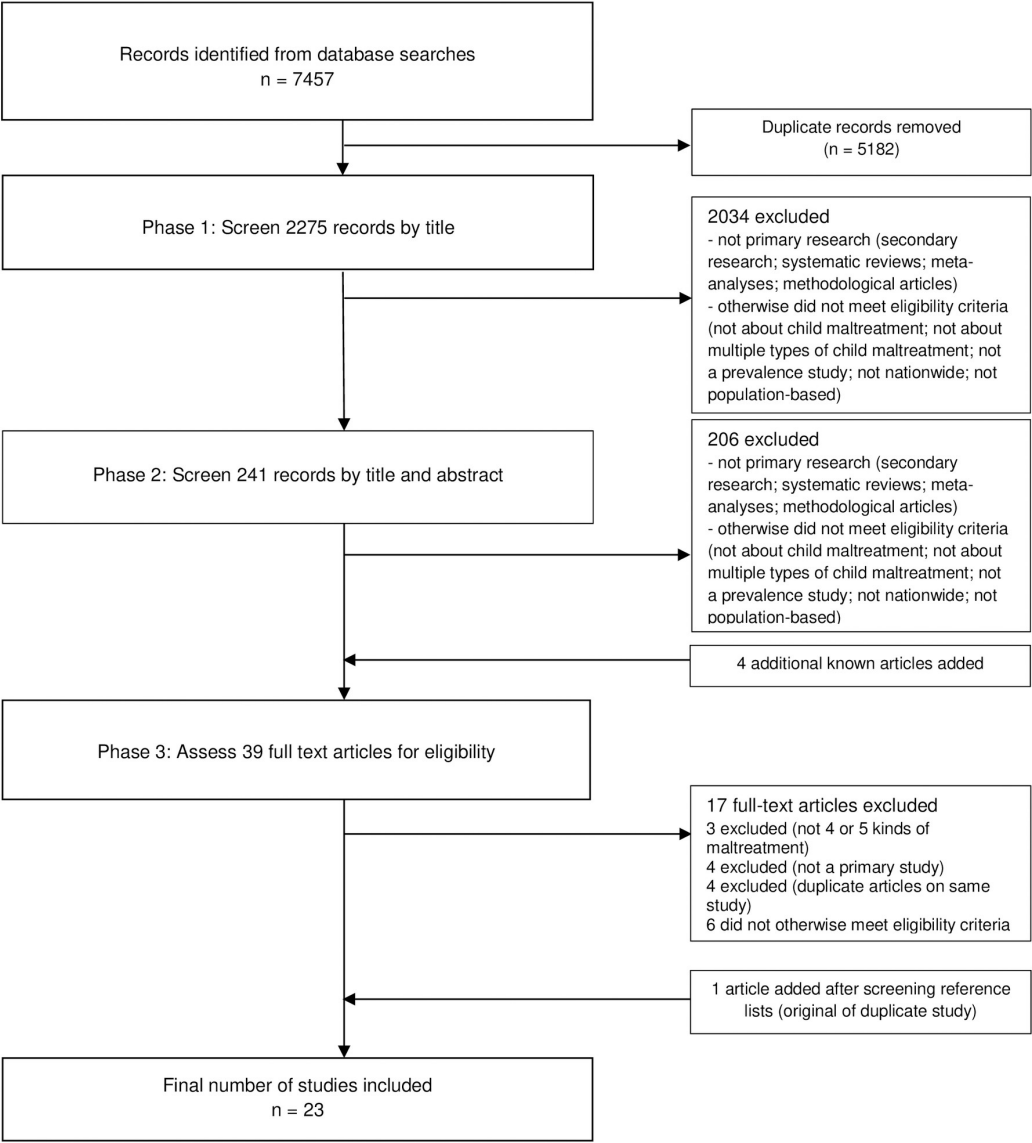
PRISMA flow diagram.

As detailed in our search strategy (S1 File), in Phase 1, MS, JD and ED screened records by title. We removed duplicates using electronic software (Endnote), and removed remaining duplicates about the same study, selecting the publication providing the most detailed account. In Phase 2, BM and RP independently screened records by title and abstract. Disagreements were discussed between BM and RP to achieve consensus. To identify any further potential eligible studies at this stage that may not have been captured in the search, all co-authors considered if there were any further known studies requiring inclusion that were not in the Phase 2 shortlist. In Phase 3, BM and RP independently assessed full text of screened in articles. Disagreements were discussed between BM and RP to achieve consensus, with reasons recorded. We screened reference lists of included studies to identify any further potential eligible studies. We used a translator to assist in screening non-English studies. This process resulted in 23 eligible studies (**Fig 1**).

### Data extraction and analysis

We developed a template to extract 60 data items from each study considering design, procedure, sample, instrument, ethics, and subpopulation analysis (S2 File). We extracted 45 items about the instrument, including: name, psychometric data, definitions of maltreatment constructs, number of questions asked about each type, and whether questions explored: (a) the relationship between the child and the person inflicting maltreatment; (b) nature of the acts; (c) severity (e.g., if they caused injuries); (d) frequency. MS and BM extracted these data. We separately tabulated the extracted items each study asked about maltreatment, with BM conducting a final double-check regarding these (S3 File).

Our critical analysis included an appraisal of the construct validity of study items and the soundness of their operational definition. To inform this analysis, we identified robust conceptual understandings of each maltreatment type as established in the scholarly literature, and adopted these as an evaluative standard. Physical abuse involves intentional acts of physical force by a parent or caregiver, excluding lawful corporal punishment [41]. Sexual abuse involves contact and non-contact sexual acts, inflicted by any adult or child in a position of power over the victim, to seek or obtain physical or mental sexual gratification, when the child does not have capacity to provide consent, or has capacity but does not provide consent [42]. Emotional or psychological abuse is inflicted by a parent or caregiver, and includes emotional unavailability, hostile interaction, developmentally inappropriate interaction, failure to acknowledge the child’s individuality, and failure to integrate the child into the social world [43]. Neglect involves parental or caregiver omissions to provide the basic necessities of life suited to the child’s developmental stage, as recognised by the child’s cultural context, including physical, emotional, medical, environmental, supervisory, and educational neglect [44]. Exposure to domestic violence involves the child witnessing a parent or other family member being subjected to assaults, threats or property damage by another adult or teenager normally resident in the household [12].

Our critical analysis was also informed by an understanding that prevalence studies must be conducted with low risk of bias to obtain reliable findings. In our analysis, we assessed study rigour, quality and practicability, and used a quality assessment tool designed to assess risk of bias in population-based prevalence studies [45, S4 File]. Using our quality assessment tool, we created an overall risk of bias score for each study which summed scores for individual items (maximum score 10). RP and CM independently assessed each study considering four external validity items and five internal validity items. Disputes were resolved through an independent third assessor (MD, BM). Our critical analysis further considered suitability of approach, considering: methodology to recruit the sample and accommodate high-risk sub-samples; administration method; instrument; soundness of conceptual constructs; ethics; and practical viability.

## Results

### Systematic review

This review identified 23 articles reporting the results of national studies of the prevalence of all five or four of the recognized forms of child maltreatment. One of these articles reported the results of a study conducted simultaneously in nine countries in the Balkan Peninsula, and eight of these national studies met our eligibility criteria [46]. Accordingly, in total, our review identified 30 national studies, conducted in 22 countries. Studies were published between 2005 and 2019. Extracted data revealed study location, scope, participants, data collection method, and instrument. **Table 1** presents the extracted information from included studies. The supporting information details the prevalence rates reported by each study (S5 File).

**Table 1:** Summary of study characteristics.

STUDY DETAILS		DESIGN				SAMPLE				PROCEDURE	
Citation	Nation	Prev/ Incid/ Both	Period studied	Number of types of maltreatment	Participant age (yrs)	Sampling frame	Sampling strategy	Sample size (n)	Response rate (%)	Recruitment	How administered^1^
**Al Muneef 2017**	Saudi Arabia	Prev	Childhood < 18	5	18 and over	Cross-sectional national sample of citizens raised and resident in S.A.	Random selection of large and small cities in all 13 regions of SA; participants selected from 182 locations	10,156	Not reported	NR	HH, hard copy (6 mths)
**Chan 2011**	Hong Kong	Both	Lifetime and past year	4: PEN EDV	12–17	Chinese children aged 12–17	Randomly sampled HHs from registry of Quarters (fixed sampling intervals); N = 4347 households	1094 (3049 HHs)	70%	NR	HH, FTF interviews
**Chan 2011**	Hong Kong	Both	Lifetime and past year	4: PEN EDV	12–17	Chinese children aged 12–17	HHs from registry of Quarters, stratified random sampling (N not reported)	2062	70%	NR	HH, FTF interviews
**Christoffersen 2013**	Denmark	Prev	PA, EA, Neg < 12; SA < 24	4: SPEN	24 yrs only	All children born 1984	Stratified random sample of 24 year olds N = 4718	2980	63%	Letter + phone	CATI + HH (2 yrs)
**Denholm 2013**	UK	Prev	Childhood < 16 yrs	5*	Age 7, 11,16, 23, 33, 42, 45, 50	All born March 1958 England, Scotland, Wales	1958 British cohort, born during one week in March (prospective cohort study). Aged 45, N = 11971	9310	78%	Birth registry	HH FTF (parent proxy for child; then direct (1 yr)
**Euser 2013**	Netherlands	Incid	Past year	5	12–17	Students aged 12–17	Random selection of 42 high schools from database of all schools nationally, each with 4 randomly selected classes	1920 (from 29/42 schools)	Not reported; 29/42 schools participated	NR	In school, hard copy
**Feng 2015**	Taiwan	Both	Lifetime and past year	5	12–18	Taiwanese adolescents aged 12–18	35 schools out of 44 invited schools, across 17 cities and townships	5236 (in 35 participating schools)	Not reported; 99·4% of participating schools	Phone, through school	In school
**Finkelhor 2005**	USA	Incid	Past 12 months	5	2–17 (parents for children 2–9; children 10–17)	Nationally representative sample children aged 2–17	Random digit dialling	2030 (1000 children 10–17; 1030 parents of children 2–9)	79·5% of eligible persons contacted	Phone	CATI (3 mths)
**Finkelhor 2009**	USA	Both	Lifetime and past 12 months	5	0–17 (parents for children 0–9; children aged 10–17)	Cross-sectional national sample of children aged 0–17	National landline residential telephone survey	4549 (3053 national cross-section; 1496 oversample)	71% cross-section; 63% oversample	Phone	CATI (5 mths)
**Finkelhor 2014**	USA	Both	Lifetime and past 12 months	5	1 mth-17 yrs (parents for those 1 mth-9 yrs; children 10–17)	Nationwide sampling frame of residential phone numbers	Random digit dialling + two samples to capture those without landlines: cell phone sample (n = 31; abandoned due to low yield) and address-based sample (n = 750)	4503	60% of eligible respondents	Phone	CATI (12 mths)
**Finkelhor 2015**	USA	Both	Lifetime and past year	5	0–17 (parents for children 0–9; children 10–17)	National sample	Nationally representative sample of phone numbers via 4 methods (address-based sample (ABS) of HH phone numbers; prescreened sample (PSS) of HH with children; landline sample (LLS); cellphone sample (CS)	4000 (1011 ABS; 520 PSS; 2443 LLS; 26 CS)	Differed across 4 sample frames (14·2%-67%)	Phone	CATI (9 mths)
**Hauser 2011**	Germany	Prev	Childhood < 18	4: SPEN	14–90	14–90 yr olds understanding written German	Cross sectional randomly generated sample of the population N = 4455	2504	56%	HH in person	HH, hard copy (1 mth)
**Lev-Wiesel 2018**	Israel	Both	Lifetime and past year	5	12–17	Students in grades 6, 8 and 10 in the national public school system	Two-stage random sample by school level (primary, junior high, high), school district (Northern, Central, Southern Israel, Jerusalem); and school SES indicator; and random selection of two classes from each grade within each school	12,035	Not reported	School	In school, either hard copy or CASI using iPod
**May-Chahal 2005**	UK	Prev	Childhood < 18	4: SPEN	18–24	18–24 year olds in the UK	Random sample using postcode address file: 633 postcode sectors with probability proportional to population of 18–24 year olds after stratification; 90 addresses in each postcode; N = 56,979 addresses	2869	69%	Direct to individual	HH, CAPI (5 mths)
**Nagy 2019**	Hungary	Prev	Childhood < 18	5	18–112	Hungarian adults aged 18 or older	Multi-stage stratified cluster sampling using 120 census sampling units with randomised selection of 10 addresses within each unit	1174	74.6%	HH in person	HH, hard copy (1 mth)
**Nikolaidis 2018**	Balkans	Both	Lifetime and past year	4: SPEN	11, 13, and 16 year olds	11, 13, and 16 year olds in nine Balkan nations	Random sample of schools, derived from number of schools per region; 63,250 students	42,194	66.7% (students); 45.8%-82.7% (nations)	School	In school, hard copy
**Radford 2013**	UK	Both	Childhood < 18, and past year	5	Parents of children 2 mths-10 yrs; Children 11–17; Adults 18–24	Children and young people in the UK aged under 25	Random probability sampling of HHs from UK Postcode Address File (50,000 by mail), and eligibility determined by visits	2160 parents of children 2 mths-10 yrs; 2275 children 11–17; 1761 adults 18–24	60.4% (number of interviews completed as a % of HHs approached)	Direct to individual by mail, door-knock	HH, CASI + option of headphones, hard copy for parent (10 mths)
**Schick 2016**	Switzerland	Prev	Childhood	4: SPEN	9th grade students	9th grade adolescents in representative population based sample	Probability proportional to cluster size, stratified by 7 regions & 26 cantons; then N = 228 randomly selected schools with 560 classes	6787 (177 participating schools with 449 classes)	RR in participating classrooms was 92%	School	In school, CASI (9 mths)
**Shen 2016**	Taiwan	Incid	Past 12 months	5	10–11	Fourth grade Taiwanese primary school children	Proportionally stratified according to county and randomly selected. N = 25% of all primary schools in Taiwan	49% of invited schools (314 schools; 6233 children)	Not reported. 99·9% of consenting parents' children participated	Phone	In school, hard copy (spring semester)
**Tsuboi 2015**	Japan	Prev	Less than 18 yrs	4: SPEN	20–49	Japanese adults aged 16–49	Multi-stage randomised cluster sampling; 44 clusters from 11 geographical units; N = 2693	1540	57·20%	Door-knock	Hard copy to home (1 mth)
**van der Kooij 2015**	Suriname	Both	12–17: childhood and past 12 mths; adults: in childhood	5	12–17; and 18–22	Suriname—national sample of students	Stratified national sample of students from high schools and vocational education classes. Random probability sampling.	1391 (57 schools); 1072 children 12–17; 239 adults 18–22	Not reported	School	In school, hard copy (4 mths)
**Ward 2018**	South Africa	Both	Lifetime and past year	5	15–17	South Africa–nationwide sample of 15–17 year olds	Multi-stage stratified random sample using 725/80,787 randomised census enumerator areas, with randomised selection of 5–10 HHs in each	5631	94.8% participation rate	HH in person	HH, hard copy interview (1 yr 5 mths)
**Witt 2017**	Germany	Prev	Childhood < 18	4: SPEN	14–94	14–94 yr olds understanding German	Randomly generated representative sample obtained by households in every third street	2487	51.20% 2510/4902 HHs	HH in person	HH, hard copy (3 mths)

CAPI: Computer assisted personal interview. CASI: Computer assisted self-interview. CATI: Computer assisted telephone interview. EDV: Exposure to domestic violence. E: Emotional or psychological abuse. HH: Household. FTF: Face to face. N: Neglect. P: Physical abuse. S: Sexual abuse.

*Each wave did not measure all five types.

^1^ Instrument administration time generally ranged from 30–55 minutes.

There were four studies in the USA [47–50], three in the UK [51–53], two in Hong Kong [54–55] two in Taiwan [56–57], and two in Germany [58,59]. There was one study in Denmark [60], the Netherlands [61], Switzerland [62], Japan [63], Suriname [64], Saudi Arabia [65], Israel [66], South Africa [67], and Hungary [68]. In the Balkans study [46], eight met eligibility criteria based on the number of types of maltreatment studied: Albania, Bosnia & Herzegovina, Bulgaria, Croatia, the Former Yugoslavian Republic of Macedonia, Greece, Romania, and Serbia; in general for our purposes, we treat these as one study. The Turkish study involved three forms of maltreatment, so was excluded from our analyses.

Fourteen studies measured all five maltreatment types [47–51,53,56–57,61,64–68]. Of nine studies measuring four maltreatment types, seven omitted EDV [46,52,58–60,62–63], and two omitted sexual abuse [54–55]. Eleven studies measured prevalence throughout childhood and in the past year; nine measured prevalence through childhood only, and three measured past year incidence only.

Only nine studies explored all five types of maltreatment across childhood, defined as aged under 18 [48–50,53,64–68]. These studies occurred in seven countries (USA, UK, Suriname, Saudi Arabia, Israel, South Africa and Hungary), and only three involved a sample of adults providing data about experiences over their entire childhood [53,65,68]. Four studies in Germany, the UK and Japan obtained information from adults about all maltreatment across childhood except EDV [52,58–59,63].

Eight studies involved only child participants aged under 18 providing self-report data. Three studies included child and adult participants each providing self-report data. Five studies involved a household’s child participant aged under 18 providing self-report data (four involved children aged 10–17 and one involved children aged 11–17) and the household’s parents providing proxy data about a child aged under the cut-off. Five studies involved only adults providing self-report data (24 year olds; 18–24 year olds; 20–49 year olds; 18 and over). Sample sizes ranged from 1094 to 12,035 participants. Five studies adopted measures to recruit high-risk sub-populations [48,56,60,62,64].

Seven studies were conducted in schools: Taiwan [56–57], the Netherlands [61]. Switzerland [62], Suriname [64], and the Balkans study [46]. Eleven studies were conducted in households by interviews, in Hong Kong [54–55], Hungary [68], the UK [51–53], Germany [58–59], Japan [63], Saudi Arabia [65], and South Africa [67]. Five studies used remote computer assisted telephone interviews (CATI), with four in the USA [47–50], and one in Denmark [60]. Data collection time ranged from 1 month to 2 years.

Methodologies to recruit the sample and accommodate high-risk subpopulations also varied. In most studies, the target population was a close representation of the national population. Studies in schools were done in countries with high school attendance. All studies used random selection. However, few studies used strategies to capture participants from culturally and linguistically diverse groups, or from high-risk groups such as those in out of home care.

Response rates for household studies generally ranged from 56% to 78%, with one reporting a participation rate of 94.8% [67]. Rates in school-based studies showed schools’ participation rate ranging from 49%-79%, and then with almost 100% response rates from children in participating schools. Response rates in CATI studies ranged from 60% to 79.5%, with more recent studies having lower rates [47–49].

Regarding consent to participate, 18 of the studies involved child participants exclusively or with adult participants. Nine studies involved only child participants; in these, two required only the child’s consent [56,62], one required the child’s consent and parental passive consent [64], one required the child’s consent and either passive or active parental consent [46], and five required parental active consent and the child’s consent [54–55,57,61,66–67].

Of the studies involving child participants, seven reported the measures used by research teams when a child was suspected to have been harmed or at risk [46–50,53,67]. Nine studies reported other measures to assist any distressed participants [46,48,52,54,56,60,62,64].

Studies used a range of instruments and approaches to measuring each maltreatment type. **Table 2** presents key data extracted from the instrument used in each study. Comprehensive details about the maltreatment items are detailed in the supporting information (S3 File).

**Table 2:** Key features of instruments used in prevalence studies.

Study	INSTRUMENT			SEXUAL ABUSE		PHYSICAL ABUSE		EMOTIONAL ABUSE		NEGLECT		EDV	
	Instrument	Psycho-metrics reported	Approach to constructs	How many items	Identity, nature, severity, frequency	How many items	Identity, nature, severity, frequency	How many items	Identity, nature, severity, frequency	How many items	Identity, nature, severity, frequency	How many items	Identity, nature, severity, frequency
Al Muneef 2017	ACE-IQ	No	S3 File	4	N N N Y	2	N N N Y	2	N N N Y	4	N N N Y	3	N N N Y
Chan 2011	CTSPC (P, E, N); CTS2 (EDV)	Yes [69–70]^1^	p. 536 [69–70]^1^	n.a	n.a	13	Y Y N Y	5	Y Y N Y	5	Y Y N Y	39	Y Y N Y
Chan 2011	CTSPC (P, E, N); CTS2 (EDV)	Yes [69–70]^1^	p. 6–8 [69–70]^1^	n.a	n.a	13	Y Y N Y	5	Y Y N Y	5	Y Y N Y	39	Y Y N Y
Christoffersen 2013	Self-developed	No	p. 152–3	4	Y Y N N	7	Y Y Y N	6	Y Y N N	7	Y Y N N	n.a	n.a
Denholm 2013	Blended tools	No	p. 342, 346	1	Y Y N N	1	Y Y N N	2	Y Y N N	11	Y Y Y N	1	Y Y N N
Euser 2013	Blended tools	No	p. 844; EDV not reported	8	Y Y N N	8	Y Y N N	1	Y Y N N	8	Y Y N N	7	Y Y N N
Feng 2015	ICAST-CH	Yes [71]	p. 12, 15	6	Y Y N Y	9	Y Y N Y	8	Y Y N Y	6	Y Y N Y	7	Y Y N Y
Finkelhor 2005	JVQ	Yes [72]^2^	p. 21–23	7 + followups	Y Y Y Y	1 + followups	Y Y Y Y	1 + followups	Y Y Y Y	1 + followups	Y Y Y Y	2 + followups	Y Y Y Y
Finkelhor 2009	JVQ (1^st^ enhanced)^3^	Yes [72]^2^	p. 1418–22	7 + followups	Y Y Y Y	1 + followups	Y Y Y Y	1 + followups	Y Y Y Y	1 + followups	Y Y Y Y	8 + followups	Y Y Y Y
Finkelhor 2014	JVQ (2^nd^ enhanced)^3^	Yes [72]^2^	p. 1433–35	7 + followups	Y Y Y Y	1 + followups	Y Y Y Y	1 + followups	Y Y Y Y	5 + followups	Y Y Y Y	8 + followups	Y Y Y Y
Finkelhor 2015	JVQ (3^rd^ enhanced)^3^	Yes [72]^2^	eApp. 2	8 + followups	Y Y Y Y	1 + followups	Y Y Y Y	1 + followups	Y Y Y Y	5 + followups	Y Y Y Y	8 + followups	Y Y Y Y
Hauser 2011	CTQ Short-form	Yes [73]	Referred to [73]	5	N Y N Y	5	N Y Y Y	5	N Y Y Y	10	Y Y Y Y	n.a	n.a
Lev-Wiesel 2018	JVQ (modified) + CTQ (modified)	Yes [72–73]^2^	Referred to [72–73]^2^	11	Y Y Y Y	6	Y Y Y Y	5	Y Y Y Y	12	Y Y Y Y	2	Y Y Y Y
May-Chahal 2005	Self-developed	No	p. 972–976	14	Y Y Y Y	9	Y Y Y Y	7	Y Y Y Y	8	Y Y Y Y	n.a	n.a
Nagy 2019	ACE	No [37]	p. 14	1	N N N N	1	N N N N	1	N N N N	2	N N N N	1	N N N N
Nikolaidis 2018	ICAST-CH (modified)	Yes, p. 5	S3 File	5 (11 yrs), 6 (13, 16 yrs)	Y N N Y	15 (11 yrs), 16 (13, 16 yrs)	Y N N Y	17 (11 yrs),19 (13, 16 yrs)	Y N N Y	4	Y N N Y	n.a	n.a
Radford 2013	JVQ (modified)	Yes [72]^2^	p. 812–3	7 + followups	Y Y N Y	2 + followups	Y Y N Y	1 + followups	Y Y N Y	14 + followups	Y Y N Y	6 + followups	Y Y N Y
Schick 2016	JVQ (modified)	Yes [72]^2^	p. 5	1	Y Y N N	1	Y Y N N	1	Y Y N N	1	Y Y N N	n.a	n.a
Shen 2016	Blended tools	Yes	p. 8–9	2	N Y N Y	7	Y Y N Y	4	Y Y N Y	4	Y Y N Y	2	Y Y N Y
Tsuboi 2015	Lifestyle & Attitudes	No	p. 2580	1	N Y N N	1	N Y N N	1	N Y N N	1	N Y N N	n.a	n.a
van der Kooij 2015	Blended tools	Yes^3^ [74–75]	p. 155 + S3 File	7	N Y N Y	8	Y Y N Y	1	Y Y N Y	8	Y Y N Y	7	Y Y N Y
Ward 2018	JVQ (modified)	Yes [72]^2^	S3 File	7	Y Y Y Y	1	Y Y Y Y	1	Y Y Y Y	4	Y Y Y Y	7	Y Y Y Y
Witt 2017	CTQ Short-form	Yes [73]	Referred to [73]	5	N Y N Y	5	N Y Y Y	5	N Y Y Y	10	Y Y Y Y	n.a	n.a

CTSPC: Conflict Tactics Scale Parent-Child version. CTS2: Conflict Tactics Scale 2. CTQ: Childhood Trauma. ICAST-CH: International Child Abuse Screening Tool: Children’s Home version. JVQ: Juvenile Victimization Questionnaire.

^1^ For further details see S3 File.

^2^ For full details on the original and subsequent enhanced JVQ, see S3 File.

^3^ For full details see S3 File.

Eight studies used the Juvenile Victimization Questionnaire (JVQ). These studies used different versions of the JVQ, either using its original form [72], an enhanced form [48–50], or an adapted version [53,62,66–67]. Two studies used the Conflict Tactics Scale Parent-Child version (measuring physical abuse, emotional abuse, and neglect), and the CTS2 (EDV) [54–55]. Two studies used the ICAST-C, in either its original form [56] or an adapted version [46]. Two studies used the Childhood Trauma Questionnaire [58–59]. Single studies used the Adverse Childhood Experiences International Questionnaire [65], the Adverse Childhood Experiences questionnaire [68], and the Lifestyle and Attitudes Towards Sexual Behavior instrument [63]. Four studies used a blend of instruments [51,57,61,64]. Two studies used self-developed instruments [52,60].

Six studies did not report psychometric data on instrument validity and reliability. Six studies reported psychometric data on the instrument as used [46,54–56,58,72]. Studies using enhanced or adapted versions of instruments generally cited the original instrument’s data but did not report further psychometric tests.

Most studies did not define overarching concepts of each form of maltreatment, instead operationalising these concepts into questions about the participant’s experiences. Approaches to some but not all forms of maltreatment broadly aligned with the nature of maltreatment concepts as established by the scholarly literature. Approaches to physical abuse and sexual abuse were generally sound. Approaches to the construct and operationalisation of emotional abuse were generally sub-optimal, with some exceptions (e.g., [46,52]). Neglect was also rarely well-operationalised, with some exceptions (e.g., [49,52–53,58–59,66].

Studies explored maltreatment experiences in varying depth, reflected by the number and nature of questions asked (**Table 2**). For sexual abuse, 12 studies asked between five and eight questions. Most studies asked about the relationship with the person inflicting the abuse, and the nature of the acts; more than half asked about frequency; but few asked about severity. Other notable differences included: two studies being limited to sexual abuse by a parent/guardian [51,60]; most studies including contact and non-contact acts, but three studies included contact abuse only [62,65,68]; four studies asking only one question [51,62–63,68].

For physical abuse, eight studies asked only one question, although these included multiple distinct concepts [47–51,62,63,68]. Six studies asked between five and nine questions. Most asked about relationship and nature; more than half asked about frequency; but few asked about severity. A notable difference was in the treatment of spanking on a child’s bottom: seven studies excluded “spanking on your bottom” from the definition of physical abuse [47–50,53,62,66]; four studies included spanking with a bare hand as physical abuse [46,54–56]; and four studies included as physical abuse being hit or spanked on the bottom but only when done with an implement or hard object [51,52,57,64].

For emotional or psychological abuse, eight studies asked between five and eight questions. Most asked about relationship and nature; more than half asked about frequency; but few asked about severity. Other notable differences included: three studies being limited to a single generic question [51,61,64]; seven studies using a single compound question [47–51,62,67]; and only two studies using a detailed scale of items closely aligned with a sound conceptual model [46,52].

For neglect, 12 studies asked between five and 11 questions. Five studies asked one question [47–48,62,63,68]. Most asked about relationship and nature; more than half asked about frequency; but few asked about severity. Six studies asked detailed questions about multiple dimensions of neglect, and their severity [49–50,52,58–59,66]. Other notable differences included: some studies operationalising neglect very broadly, including a parent having low aspirations [51], or not helping with homework [64]; only one study asking about educational neglect [64]; and one study omitting physical and nutritional neglect [46].

For exposure to domestic violence, six studies asked between six and eight questions. Most asked about relationship and nature; more than half asked about frequency; but few asked about severity. Notable differences were: two studies used the comprehensive CTS2 scale of 39 items originally devised for use with adult couples [54–55]; and the original JVQ had two physical assault items [72], and later added six items about threats or property damage by other family members [48–50].

### Risk of bias

**Table 3** sets out the quality assessment and scoring results for each study. Scores ranged from 6 to 10. Most studies had relatively high internal and external validity. We concluded that studies scoring 9.5 or 10 had minimal bias. Five studies achieved scores of 10: two in Hong Kong [54–55], and one each in Taiwan [56], Israel [66] and South Africa [67]. Five studies achieved scores of 9.5: three in the USA [48–50], one in the UK [53], and the Balkans study [46]. Five other studies achieved scores of 9, from Saudi Arabia [65], the UK [52], Germany [62], Hungary [68], and Taiwan [57]. Four studies scored 7, and two scored 6; here we concluded risk of bias was high, particularly regarding selection bias and non-response bias.

**Table 3:** Quality assessment of included studies.

		Quality assessment criteria									
Study	Location	Target population nationally representative (age, sex)	Representative sampling frame	Random selection	Non-response bias	Data collected from participant	Questions congruent with conceptual understandings of maltreatment	Instrument reliability and validity	Consistent data collection	Appropriate numerators, denominators	Quality score
Al Muneef 2017	Saudi Arabia	2	1	1	1	1	1	0	1	1	9
Chan 2011	Hong Kong	2	1	1	1	1	1	1	1	1	10
Chan 2011	Hong Kong	2	1	1	1	1	1	1	1	1	10
Christoffersen 2013	Denmark	1	1	1	1	1	0	0	1	1	7
Denholm 2013	UK	1	0	1	1	1	0	0	1	1	6
Euser 2013	Netherlands	1	0	1	1	1	1	0	1	1	7
Feng 2015	Taiwan	2	1	1	1	1	1	1	1	1	10
Finkelhor 2005	USA	2	1	1	1	0·5	0	1	1	1	8·5
Finkelhor 2009	USA	2	1	1	1	0·5	1	1	1	1	9·5
Finkelhor 2014	USA	2	1	1	1	0·5	1	1	1	1	9·5
Finkelhor 2015	USA	2	1	1	1	0·5	1	1	1	1	9·5
Hauser 2011	Germany	1	1	1	0	1	0	1	1	1	7
Lev-Wiesel 2018	Israel	2	1	1	1	1	1	1	1	1	10
May-Chahal 2005	UK	2	1	1	1	1	1	0	1	1	9
Nagy 2019	Hungary	2	1	1	1	1	0	1	1	1	9
Nikolaidis 2018	Balkans	2	1	1	0.5	1	1	1	1	1	9.5
Radford 2013	UK	2	1	1	1	0·5	1	1	1	1	9·5
Schick 2016	Switzerland	2	1	1	1	1	0	1	1	1	9
Shen 2016	Taiwan	2	1	1	0	1	1	1	1	1	9
Tsuboi 2015	Japan	2	1	1	0	1	0	0	1	1	7
van der Kooij 2015	Suriname	1	0	1	0	1	1	0	1	1	6
Ward 2018	South Africa	2	1	1	1	1	1	1	1	1	10
Witt 2017	Germany	2	1	1	0	1	0	1	1	1	8

## Discussion

This systematic review identified 30 studies of the prevalence of either four or five forms of child maltreatment, conducted in 22 nations. In addition, many other studies have been conducted of three or fewer maltreatment types, such as studies of sexual, physical and emotional abuse. These have been conducted on a stand-alone basis [76], or as part of a systematic campaign supported by a global public private partnership [77]. By 2019, the Violence Against Children Surveys (VACS), which also measure the prevalence of physical, sexual and emotional abuse, had been conducted in 16 countries and were being planned in a further eight countries in Africa, Asia and the Caribbean [30,77–78]. Other studies have considered the prevalence of a mixture of peer violence and maltreatment by parents or caregivers [79–80]. Accordingly, a good deal of evidence has been generated about the prevalence of child maltreatment in several dozen nations, representing substantial progress in the international understanding of the epidemiology of child maltreatment. However, this review has highlighted the fact that the vast majority of nations lack reliable benchmark national prevalence data on a comprehensive assessment of maltreatment, including measurement of four or five of the recognised five types of maltreatment, and almost all lack follow-up studies to establish trends over time. This study demonstrates the urgent need to conduct more rigorous prevalence studies, particularly those by measuring all relevant types of maltreatment, to generate more accurate understandings of the extent of maltreatment, and to enable progress in reducing child maltreatment against the SDG target.

Our review also shows that there is substantial variation in study participants across the different studies, limiting comparability and introducing certain strengths and limitations which are important to consider in designing future work. Several studies obtained data using parents as proxies for children under 10, and reported reliable responses. This approach may capture data about very young children’s experiences that is otherwise unattainable, although accurate estimates rely on parents being both knowledgeable and truthful in their responses [47]. Yet, the literature reports no evidence of reporter bias in comparisons of adult proxy and youth self-report data [47,48].

Arguably, from a public health perspective, studies provide most comprehensive and reliable estimates when capturing prevalence data over the entire span of childhood up to age 18. Furthermore, where a study’s participants are children and or young adolescents, past year incidence data is useful. Over half of the studies in this review included children as respondents. In these studies, responses benefitted from being direct and proximate to the experience as well as capturing useful stratified data about single year incidence in a closely contemporaneous time period. Developmental evidence suggests children’s and adolescents’ participation is entirely appropriate. While adolescents may generally differ from adults in the attainment of psychosocial capacities to understand long-term consequences, regulate conduct, and withstand social and emotional pressures, their cognitive capacity is not substantially different from that of adults [81–84]. Similarly, apart from those still in early developmental stages, children’s cognition and reliable episodic memory is sufficiently developed to enable participation in survey research [85–86]. This justifies the design of instruments for child and adolescent participants, including the careful approach of the developers of the Juvenile Victimization Questionnaire in designing an instrument suitable for participants as young as eight [72].

Ethically, there is no impediment to involving child and adolescent participants [87]. Adolescents and children are cognitively capable of providing their own consent, and are ethically entitled to do so as autonomous individuals. Moreover, adolescents and children have rights to freedom of expression, and bear the right of participation in matters affecting them. While there remains no consensus on the most justifiable approach to confidentiality and welfare [87–90], we assert that studies can adopt robust measures to balance imperatives of attaining sufficient study participation, while ensuring participant welfare and confidentiality. While confidentiality is a foundational principle in these studies, the exception to this, conveyed to youth participants at the outset, that cases of current or imminent significant risk of danger may be referred to welfare authorities, has been found not to affect response rates [38,53]. Alongside this, studies can adopt stepwise approaches drawing on multiple psychological and legal resources to support participants who disclose severe incidents or who experience distress [87]. However, it is important not overstate the frequency of distress. Several studies have found low rates of distress among youth participants in studies of maltreatment, and the level of youth distress does not differ significantly from that of adults. Furthermore, even distressed participants mostly maintain their involvement was worthwhile [38,91]. A recent US study, for example, found only 0.8% of participants aged 10–17 reported being “pretty or a lot” upset by answering the questions, and even this did not unduly affect their reported willingness to participate [91]. An associated finding is that children in high-risk sub-populations, such as those in out-of-home care, have not been well represented, leading to likely underestimates of prevalence and scarce evidence about specific risk profiles.

Studies that rely on adults’ retrospective accounts offer the substantial benefit of capturing data about experiences across childhood. One limitation of such studies is that they will not obtain recent proximal data of single year incidence. An additional potential limitation, yet to be fully analysed, may be that retrospective accounts are affected by various kinds of recall bias. We acknowledge that some have argued that retrospective studies do not provide data about child abuse experiences that is as accurate as prospective studies [92–93] and have cautioned against sole reliance on retrospective accounts, especially where prevalence estimates are used to draw causal inferences about the effect of maltreatment on biomedical diseases. A recent systematic review and meta-analysis concluded that prospective and retrospective measures of childhood maltreatment identify different groups of individuals [94]. However, it was also recognised that prospective measures may have lower sensitivity than retrospective measures of the experience of maltreatment, and concluded that “the low agreement between prospective and retrospective measures cannot be interpreted to directly indicate poor validity of retrospective measures” and that retrospective measures could have greater ability to identify true cases [94]. The well-known discrepancies between true maltreatment rates and those recorded in many data sources used for prospective studies is attributable to the low correlation between actual experiences and their representation in official data such as crime statistics and child protection service records. Few maltreatment experiences are ever brought to the attention of criminal justice agencies or child protection services. The caution urged regarding retrospective reports appropriately appears more directed towards studies considering causation of disease than estimation of population prevalence. It is also accepted that lack of validity tends to underreport the experience of abuse [95–97], and studies of test-retest reliability regarding retrospective accounts have indicated general stability over time [98]. We acknowledge that retrospective reports may have compromised validity for various reasons, including motivational factors and memory biases, and measurement features including poorly worded questions [92,94]. Overall, however, our view is that retrospective studies of child maltreatment, especially when well-designed with behaviourally-specific questions grounded in sound constructs of maltreatment, with representative samples of the population, offer the opportunity to obtain sufficiently accurate estimates of the prevalence of child maltreatment experiences.

The fourth finding is that while considerable investment is required for all kinds of approach, viable approaches to survey administration are available for diverse geographical settings to accommodate large and small nations, and attain sufficient participation. The implications of this are clear for future study design. School-based studies appeared most often in small nations, which may more readily facilitate centralised educational sector endorsement for the research, or which may have a high commitment to social research. When school leaders agree for their school to participate, children generally participate at a very high rate. Similarly, household studies identified in this review generally occurred in small nations. Both school-based and household studies require substantial numbers of staff, but may be most feasible where labour costs are manageable and where the social ecology is of sufficient strength to support and perhaps even require direct personal involvement in such research. In larger nations, for reasons of practicability and cost, studies used CATI and achieved satisfactory response rates. Perhaps for reasons of cost, and practical difficulty, a challenge remains to capture the experience of culturally and linguistically diverse sub-populations, and hard to reach groups such as children who are not in school, or who are in out of home care. Future research could consider optimal local strategies to respond to this challenge.

Our fifth finding is that selection, design and testing of an appropriate instrument appears an enduring challenge. In this regard, two coexisting needs must be balanced by any study: first, to be practicable in terms of the time and cost required to design, test and administer an instrument and minimise missing data; and second, to achieve sufficient comprehensiveness and ensure construct validity by describing maltreatment types in a way congruent with conceptual understandings [33]. Our review showed that a wide variety of instruments have been used, with psychometric data often not reported. The JVQ was the instrument most often used in either full-form or short-form; moreover, several studies adapted the original JVQ, sometimes adding a considerable number of items. These adapted versions did not appear to have been subjected to testing. While inconsequential modification of a proven instrument obviates the need for re-testing, substantial modification may be further supported by cognitive testing and test-retest reliability. What is relatively clear is that a proven, sound instrument offers both practicable and methodological benefits over a blended tool, or a new unproven instrument.

Our sixth finding is that instruments must soundly operationalise constructs of each maltreatment type by describing them in a way congruent with sound conceptual understandings. This review and critical appraisal found that instruments most often adopted unsound constructs and operationalisation of neglect, and emotional abuse. In particular, many studies did not consider sufficient operational categories of these maltreatment types as required by sound conceptual models, which will lead to under-estimates of prevalence, and will miss the opportunity to capture important information about the nature of specific experiences. Other studies used broad or vague conceptual expressions, which will have the opposite effect of over-estimating prevalence. This finding provides a contextual demonstration of the problem of unsound constructs compromising reliability and validity in general [33,34], and of the ongoing challenge to this field to adopt sound constructs of maltreatment and sound behaviourally-specific examples of these constructs [99]. Additionally in this regard, many studies asked only one question about a maltreatment type, which leads to underestimates of prevalence [36]. Single-item assessment, even through a compound question involving multiple elements of a construct, cannot capture accurate or nuanced data and should be avoided wherever possible. Finally, we found few questions about educational neglect. Arguably, since education is a human right recognised by the United Nations Convention on the Rights of the Child article 28, and is a condition for human flourishing [100] and a protective factor against multiple adversities such as child marriage [101], this is a significant dimension of neglect warranting greater priority. We recommend particularly close attention to how future studies conceptualise and operationalise these forms of maltreatment.

A seventh finding is that few studies asked detailed follow-up questions about the child’s relationship with the person inflicting the acts, and the severity and frequency of the acts. Generally, studies using the JVQ asked the most detailed follow-up questions. Obtaining information about the severity, frequency, timing, and relational setting of abuse and neglect is important, since the closeness of the relationship between the person maltreating the child and the child can have significant effects [102–103], and the timing of maltreatment is also important, with studies finding effects for both sex and age [104]. From a public health perspective, the measurement of maltreatment should ideally move beyond raw prevalence, and yield sufficiently sensitive and nuanced information about these key contextual features of the maltreatment to inform future public health policy and prevention efforts, including the indication of priority areas for responses. The addition of such questions presents challenges for instrument design and implementation, including the time to administer additional questions. However, we recommend such questions wherever possible.

### Limitations

We reviewed studies measuring the traditional forms of child maltreatment, and excluded studies of adverse childhood experiences conceptualised more broadly, such as peer bullying and community violence. Some researchers recommend that studies include both maltreatment and these other adversities [37] on the basis that chronic exposure to multiple adversities influences developmental trajectories through the lifespan. However, we applied rigorous eligibility criteria of four or five of the recognized maltreatment categories, all clearly associated with adverse sequelae, and which most closely reflect specific SDG targets of caregiver abuse and any sexual violence. Recent outcomes of the ACE study itself have only focused on these five types and three classes of household dysfunction [18]. Additionally, our data extraction method for the quality assessment was not formally validated, but we adopted an approach similar to that used elsewhere [32,35,45] considering key variables in detail. Similarly, while there were no previously validated risk of bias measures for this specific type of prevalence study, we used a method with high interrater agreement that has been used elsewhere [45], including in prevalence studies of abuse and interpersonal violence [105–106]. Our approach to risk of bias adopted a conservative approach, and reasonably concluded that studies scoring 9.5 or 10 had minimal bias.

## Conclusions

This systematic review and analysis has shown nationwide studies of the prevalence of child maltreatment have been conducted, using methods of administration suited to the setting, and involving child participants, adult participants, or both. However, there are few such nationwide studies of all five or even four maltreatment types, leaving substantial gaps in knowledge about the experience of childhood maltreatment in nearly all countries. Overall, our review and analysis indicates many of the completed studies are generally sound, but some take a more comprehensive and conceptually robust approach to provide nuanced, useful data for researchers and policymakers. To enable measurement of progress against the United Nations Agenda for Sustainable Development Goal 16 of reduction of child abuse, many countries need to invest in robust national prevalence studies. Such studies should measure exposure to domestic violence in addition to physical abuse, sexual abuse, emotional abuse, and neglect. Studies should use an instrument with demonstrated validity and reliability, and must ensure maltreatment types are operationalised appropriately in the questions asked. If participants are children or adolescents under age 18, studies should capture past year incidence, as well as childhood prevalence. Information should be captured about the specific nature, severity and frequency of the maltreatment, and the relationship of the child to the person who inflicted the acts. Such data can best inform the development and monitoring of nationwide prevention efforts.

## Supporting information

S1 Fig PRISMA checklist. (DOCX)

S1 File Search strategy. (DOCX)

S2 File Data extraction template. (DOCX)

S3 File Extracted survey items. (DOCX)

S4 File Quality assessment tool. (DOCX)

S5 File Prevalence rates of included studies. (DOCX)
