# Supplementary material for: Improving measurement of child abuse and neglect: A systematic review and analysis of national prevalence studies
Source: PLoS One. 2020 Jan 28;15(1):e0227884. doi: 10.1371/journal.pone.0227884 (PMC6986759; doi:10.1371/journal.pone.0227884)
Supplement: S1 File — (DOCX) [file pone.0227884.s002.docx]

**S1 File. Search strategy**

We searched for peer-reviewed and grey literature presenting nation-wide empirical studies of the prevalence of either four or five forms of child maltreatment, whether including consideration of nature and context or not.

**Search approach**

The search approach had three components with search terms and operators designed to capture relevant literature and exclude irrelevant literature. The precise strategies were adapted to suit the different databases (see below for technical detail). The search was conducted using title and abstract only (i.e, not full-text).

Line 1 nation* OR population*

AND

Line 2 survey OR study

AND

Line 3 prevalence OR incidence

AND

Line 4 child*

AND

Line 5 abuse OR maltreat*

**Table 1 Systematic literature review: Database search strategy**

| **Database search strategy details** | **Endnote total** |
| --- | --- |
| **PubMed**  (((((nation*[Title/Abstract] OR population*[Title/Abstract])) AND (survey[Title/Abstract] OR study[Title/Abstract])) AND (prevalence[Title/Abstract] OR incidence[Title/Abstract])) AND child*[Title/Abstract]) AND (abuse[Title/Abstract] OR maltreat*[Title/Abstract])  Filters: publication date (to 31/05/2019), Search by title/abstract | **1202** |
| **ProQuest**  ab(nation* OR population*) AND ab((survey OR study)) AND ab((prevalence OR incidence)) AND ab(child*) AND ab((abuse OR maltreat*))  Filters: publication date (to 31/05/2019), Search by abstract, peer reviewed, document type (article) | **1128** |
| **EBSCOHost (CINHL; ERIC; MEDLINE; PsychINFO; Violence & Abuse Abstracts)**  AB ( nation* OR population* ) AND AB ( survey OR study ) AND AB ( prevalence OR incidence ) AND AB child* AND AB ( abuse OR maltreat* )  Filters: publication date (to 31/05/2019), Search by abstract, source type (Academic journals, journal articles, reports)  CINAHL: 350  ERIC: 129  MEDLINE: 1121  PsychINFO: 769  V&A Abstracts: 92 | **2461** |
| **Embase**  nation*:ab,ti OR population*:ab,ti AND (survey:ab,ti OR study:ab,ti) AND (prevalence:ab,ti OR incidence:ab,ti) AND child*:ab,ti AND (abuse:ab,ti OR maltreat*:ab,ti)  Filters: publication date (<1966 to 2019), search by title/abstract, source type (article, review) | **1149** |
| **ScienceDirect**  (nation OR population) AND (survey OR study) and (prevalence OR incidence) AND (child) AND (abuse OR maltreat)  Filters: publication date (to 2019), search by title/abstract/keywords, article type (review articles, research articles) | **242** |
| **Cochrane**  (nation* OR population*) AND (survey OR study) AND (prevalence OR incidence) AND child* AND (abuse OR maltreat*)  Filters: publication date (to 31/05/2019), search by abstract, Cochrane Reviews | **6** |
| **WHO Global Index Medicus (AIM, LILACS, IMEMR, IMSEAR, WPRIM, WHOLIS)**  (nation* OR population*) AND (survey OR study) AND (prevalence OR incidence) AND child* AND (abuse OR maltreat*)  Filters: publication date (all years to 2019), title/abstract/subject | **395** |
| **Ovid Medline (R)**  ((nation* or population*) and (survey or study) and (prevalence or incidence) and child* and (abuse or maltreat*)).ab.  Filters: publication date (1902 to 2019), abstract | **866** |
| **KoreaMed**  ( nation* [ALL] OR population* [ALL] ) AND ( survey [ALL] OR study [ALL] ) AND ( prevalence [ALL] OR incidence [ALL] ) AND child* AND [ALL] ( abuse [ALL] OR maltreat* [ALL] )  ((((TIAB:"nation* or population*") AND TIAB:"survey or study") AND TIAB:"prevalence or incidence") AND TIAB:"child*") AND TIAB:"abuse or maltreat*" | **0** |
| **Open Grey**  (nation* OR population*) AND (survey OR study) AND (prevalence OR incidence) AND child* AND (abuse OR maltreat*) | **5** |
| **Campbell Collaboration**  nation OR population AND survey OR study AND prevalence OR incidence AND child AND abuse OR maltreat | **3** |
| **African Journals Online**  (nation* OR population*) AND (survey OR study) AND (prevalence OR incidence) AND child* AND (abuse OR maltreat*) | **0** |
| **Turkish Medline**  (nation* OR population*) AND (survey OR study) AND (prevalence OR incidence) AND child* AND (abuse OR maltreat*) | **0** |

Notes: The years searched across databases vary because the databases were established at different times. We searched from inception to 31 May 2019. KoreaMed and African Journals Online searches resulted in zero results; these were in English. IndMed and IranMedex were inaccessible and we treated these as 0. The Turkish Medline search template defaults to Turkish, but can be displayed in English. As an additional secondary backup method to ensure we captured any relevant records in Turkish Medline, we conducted an additional simpler search (in both English and Turkish) to broaden the search, using a search string of the terms “child” and “abuse”. This produced 4 records, none of which were relevant.

**Identification of studies**

The review resulted in 7457 records from all searches combined. We removed 5182 duplicates using electronic software (Endnote) and CI input, resulting in a total of 2275 distinct records. These were then screened in a three phase process as detailed in our article and PRISMA flow diagram, resulting in the identification of 23 eligible studies. Inclusion and exclusion criteria are shown in Table 2.

**Table 2 Systematic literature review: Inclusion and exclusion criteria**

| **Dimension** | **Included studies** | **Excluded studies** |
| --- | --- | --- |
| Type of research | Original empirical research (primary research) | Secondary research |
| Publication type | Peer-reviewed research published in scholarly refereed journals, and grey literature |  |
| Overall nature of the study | Studies of the prevalence or incidence of four or five types of maltreatment in childhood (physical abuse, sexual abuse, psychological or emotional abuse, neglect, and exposure to domestic violence) and of the nature and context of the CM (perpetrator; nature, severity, frequency or duration of CM) | Studies of other broader kinds of childhood adversity such as bullying, exposure to community violence e.g. street crime, exposure to or involvement in civil conflict, trafficking, specific cultural violence e.g. female genital cutting/mutilation  Studies only of contextual factors e.g. disclosure, health consequences |
| Parameters of maltreatment and perpetrator type/responsible agent | Prevalence or incidence studies of relevant forms of maltreatment by relevant perpetrator type, namely, CSA by anyone; other forms of maltreatment by parents, caregivers or institutional authorities | Studies of adversity involving other perpetrators/responsible agents e.g. bullying or psychological ‘abuse’ by peers; neglect by government agencies |
| Participant type | Studies using participant self-reporting or parent/caregiver reporting | Studies using informants i.e. other individuals or agencies |
| Sample strategy | Quantitative studies using a population-wide representative sample across an entire nation | Qualitative studies; regional or other non-nation-wide studies; other small quantitative studies using a convenience sample clinical sample or an institutional sample |
| Location | Studies conducted in any jurisdiction (includes English speaking and non-English speaking) | None |
| Time period | Studies published from database inception to 31 May 2019 | None |
