# Supplementary material for: Improving measurement of child abuse and neglect: A systematic review and analysis of national prevalence studies
Source: PLoS One. 2020 Jan 28;15(1):e0227884. doi: 10.1371/journal.pone.0227884 (PMC6986759; doi:10.1371/journal.pone.0227884)
Supplement: S2 File — (DOCX) [file pone.0227884.s003.docx]

**S2 File.**  **Data extraction template: key variables and information obtained from eligible studies**

**Details of study**

1. Author names and publication citation

2. Jurisdiction/s

**Design**

3. Was the study of prevalence (i. e. in childhood), incidence (e.g., previous year), or both?

4. How many and which of the CM types were studied (4 or 5; PA SA EA/PA N EDV)

5. What was the participants’ age range (e.g., children < 18; children and adults together; adult age range)

**Procedure**

6. What method was used to recruit the sample? (e.g. phone, school, home, online)

7. Were there measures to recruit special subgroups of participants (e.g. CALD, out of home care, institutional, disabled participants (y/n)

8. What method was used to collect data (CATI (computer-assisted telephone interview); phone, household, school, institution: P; H; S; I )

9. How long did data collection time take? (n = yrs/months; timeframe)

**Sample**

10. What was the sampling frame?

11. What was the sampling strategy?

12. What was the total sample size (n = )

13. What was the response rate (n %)

**Instrument**

14. Specific instrument used (instrument name)

15. Does the article report the instrument’s validation procedure? (y/n)

16. Does the article report the instrument’s reliability data? (y/n)

17. How did the instrument define key maltreatment terms?

a. PA was defined as ……………

b. SA was defined as ……………

c. E/PA was defined as ……………

d. N was defined as ……………

e. EDV was defined as ……………

18. Did the instrument contain specific items about PA? (yes/no). If no, go to next question. If yes:

a. How many items? (n = )

b. Were there items about perpetrator’s identity/relationship with victim? (y/n)

c. Were there items about the nature of the abuse (i.e., specific acts committed)? (y/n)

d. Were there items about the severity of the abuse? (y/n)

e. Were there items about the, frequency the abuse? (y/n)

f. Were there items about the duration of the abuse? (y/n)

19. Did the instrument contain specific items about SA (y/n). If no, go to next question. If yes:

a. How many items? (n = )

b. Were there items about perpetrator’s identity/relationship with victim? (y/n)

c. Were there items about the nature of the abuse (i.e., specific acts committed)? (y/n)

d. Were there items about the severity of the abuse? (y/n)

e. Were there items about the frequency the abuse? (y/n)

f. Were there items about the duration of the abuse? (y/n)

20. Did the instrument contain specific items about EA (y/n). If no, go to next question. If yes:

a. How many items? (n = )

b. Were there items about perpetrator’s identity/relationship with victim? (y/n)

c. Were there items about the nature of the abuse (i.e., specific acts committed)? (y/n)

d. Were there items about the severity of the abuse? (y/n)

e. Were there items about the frequency the abuse? (y/n)

f. Were there items about the duration of the abuse? (y/n)

21. Did the instrument contain specific items about N (y/n). If no, go to next question. If yes:

a. How many items? (n = )

b. Were there items about perpetrator’s identity/relationship with victim? (y/n)

c. Were there items about the nature of the neglect (i.e., specific acts/omissions)? (y/n)

d. Were there items about the severity of the neglect ? (y/n)

e. Were there items about the frequency the neglect ? (y/n)

f. Were there items about the duration of the neglect ? (y/n)

22. Did the instrument contain specific items about EDV (y/n). If no, go to next question. If yes:

a. How many items? (n = )

b. Were there items about perpetrator’s identity/relationship with victim? (y/n)

c. Were there items about the nature of the EDV (i.e., specific acts committed)? (y/n)

d. Were there items about the severity of the EDV ? (y/n)

e. Were there items about the frequency the EDV ? (y/n)

f. Were there items about the duration of the EDV ? (y/n)

23. Did the instrument ask the participant if she or he disclosed the abuse or neglect, and if so, to whom? (y/n, information about receiver of disclosure)

**Ethics**

24. If the study involved participation by children, was consent obtained from the adult, the child, or both? (adult, child, both)

25. For studies involving child participants, were there measures put in place to report suspected current abuse or neglect? (y, n, not reported)

26. Were there measures put in place to provide support to participants in case of distress? (y, n, not reported)

**Analysis**

27. Were there strategies for special subgroups e.g. ATSI, CALD, institutional e.g. by weighting, oversampling, clustering (yes/no)
