# Supplementary material for: Improving measurement of child abuse and neglect: A systematic review and analysis of national prevalence studies
Source: PLoS One. 2020 Jan 28;15(1):e0227884. doi: 10.1371/journal.pone.0227884 (PMC6986759; doi:10.1371/journal.pone.0227884)
Supplement: S3 File — (DOCX) [file pone.0227884.s004.docx]

**S3 File. Extracted survey items about child maltreatment experiences**

Here, we list the items extracted from survey instruments about experiences of the five forms of abuse and neglect. Some instruments (notably the JVQ) included items on child maltreatment as conceptualized in this article, as well as other items (such as on community violence, and peer bullying). In these cases, we extracted those items that most closely, conservatively and indisputably represent the five forms of abuse and neglect as we conceptualised them for the purpose of this article, as specified in the protocol and as reported in the article (see the Introduction, the inclusion criteria explained in the methods section, and our reference to conceptual models of these forms of maltreatment in the discussion). So, for example, we did not extract items on experiences that are not considered to be components of any of these forms of abuse (e.g., exposure to community violence; exposure to parental alcohol abuse; school bullying). We did extract items on:

- physical abuse, emotional or psychological abuse, and neglect, as inflicted by parents, caregivers or institutional authority figures;
- exposure to domestic violence or family violence or intimate partner violence (as these concepts are understood in the literature);
- sexual abuse by anyone.

We extracted items from each study as reported in the published article. Some studies did not include the instrument in full. Where possible in these cases, we located the instrument by identifying the publication in which it was detailed (or the actual instrument itself if located in some other place), and extracted the items used by the authors of the study. These additional publications are detailed below. In three cases this was not possible; here, we contacted the authors and requested the instrument. In two of these cases we were not able to obtain the instrument, and had to rely only on what was reported in the article.

As shown in the article, the 23 studies used one of the following instruments:

1. **Juvenile Victimization Questionnaire** (eight studies, in either original form: Finkelhor 2005; or enhanced form: Finkelhor 2009, 2014, 2015; or an adapted version of the JVQ: Lev-Wiesel 2018; Radford 2013; Schick 2016; Ward 2018)

2. **CTSPC and CTS2** (two studies: Chan 2011, Chan et al. 2011)

3. **ICAST-CH** (two studies, in either original form: Feng 2015; or modified form: Nikolaidis 2018)

4. **Childhood Trauma Questionnaire** (two studies: Hauser 2011; Witt, 2017)

5. **Lifestyle and Attitudes Towards Sexual Behavior** (one study: Tsuboi 2015)

6. **Self-developed tools** (two studies: Christofferson 2013; May-Chahal 2005)

7. **Blended tools** (four studies: Denholm 2013; Euser 2015; Shen 2016; Van der Kooij 2015)

8. **Adverse Childhood Experiences International Questionnaire** (one study: Al Muneef 2017)

9. **Adverse Childhood Experiences** (one study: Nagy 2019)

**1. Juvenile Victimization Questionnaire Studies**

Eight studies used the JVQ in either its original form, its enhanced form, or an adapted form.

1. **Finkelhor 2005** used the original form of the JVQ, including the following:
   1. SA (S1-S7);
   2. PA (M1);
   3. E/PA (M2);
   4. Neglect (M3); and
   5. EDV (W1, W2).

The original JVQ (Finkelhor et al. 2005) collected data on 34 experiences in modules representing five victimization types (conventional crime; child maltreatment; peer and sibling victimization; sexual victimization; witnessing and indirect victimization; the JVQ can be used in full or in modules). The 34 items were “screener” items, and if a participant had experienced an event, follow-up questions obtained further data: p. 407. We extracted screeners matching maltreatment types, sexual victimization, and EDV. Subsequent versions of the JVQ were not reported as being subject to psychometric tests. The first enhanced JVQ had 48 screeners; the notable difference is an extra six EDV screeners. The second enhanced JVQ was discussed elsewhere (Finkelhor et al. 2013); the notable difference is an extra four neglect screeners. The third enhanced JVQ (Finkelhor et al. 2015) has one added item on sexual abuse. Adapted versions of the JVQ are either still comprehensive (Radford et al. 2013; Lev-Wiesel et al. 2018; Ward et al. 2018) or considerably shorter: Schick et al. (2016) used selected items from the JVQ to measure child maltreatment “as proposed by the authors” of the JVQ in earlier work (Hamby et al 2005), but did not use follow-up items, and sexual abuse was limited to contact abuse by known adults.

1. **Finkelhor 2009** used an enhanced version of the JVQ which included all the 2005 items noted above, and added:
   1. Six new items for EDV (EF1-EF6)
2. **Finkelhor 2014** used a further enhanced version of the JVQ which included all the 2005 items noted above, and the six new EDV items from 2009, and also added:
   1. Four new items for Neglect (M5, M6, M8, M9).
3. **Finkelhor 2015** used:
   1. a further enhanced version of the JVQ which included all the 2005 items noted above, the six new EDV items from 2009, the four new Neglect items from 2014; and
   2. one new item for SA (S8).
4. **Radford 2013** used a modified version of the JVQ, using:
   1. SA (S1, S3, S4, S5, S6, S7) and a new item;
   2. PA (M1) and a new item;
   3. E/PA (M2);
   4. Neglect (a wide range of items, one (M3) from the JVQ, and the others being new); and
   5. EDV (W1, W2, EF1, EF2, EF5, EF6).

Note also that Radford’s study excluded data for children aged under 3.

1. **Schick 2016** used a modified version of the JVQ, using:
   1. SA (S1);
   2. PA (M1);
   3. E/PA (M2); and
   4. Neglect (M3).
2. **Lev-Wiesel 2018** used a modified version of the JVQ, using:
   1. SA (S1, S2, S3, S4, S5, S6);
   2. PA (M1);
   3. E/PA (M2);
   4. Neglect (M3);
   5. EDV (W1, W2); and
   6. The Childhood Trauma Questionnaire as used in Hauser 2011, described below (5 items each for SA, PA, E/PA, and 10 items for neglect).
3. **Ward 2018** used a modified version of the JVQ, using:
   1. SA (S1, S2, S3, S4, S5, S6, S7);
   2. PA (M1);
   3. E/PA (M2); and
   4. Neglect (M5, M6, M8, M9); and;
   5. EDV (W2, EF1, EF2, EF3, EF4, EF5, EF6).

**Screener items from the Original JVQ**

**Physical abuse (1)**

M1) Physical Abuse by Caregiver

Not including spanking on your bottom, at any time in your life, did a grown-up in your life hit, beat, kick, or physically hurt you in any way?

**Psychological/emotional abuse (1)**

M2) Psychological/Emotional Abuse

At any time in your life, did you get scared or feel really bad because grown-ups in your life called you names, said mean things to you, or said they didn’t want you?

**Neglect (1)**

M3) Neglect

When someone is neglected, it means that the grown-ups in their life didn’t take care of them the way they should. They might not get them enough food, take them to the doctor when they are sick, or make sure they have a safe place to stay. At any time in your life, were you neglected?

**Sexual abuse (7)**

S1) Sexual Assault by Known Adult

At any time in your life, did a grown-up you know touch your private parts when they shouldn’t have or make you touch their private parts? Or did a grown-up you know force you to have sex?

S2) Sexual Assault by Unknown Adult

At any time in your life, did a grown-up you did not know touch your private parts when they shouldn’t have, make you touch their private parts or force you to have sex?

S3) Sexual Assault by Peer/Sibling

Now think about other kids, like from school, a boy friend or girl friend, or even a brother or sister. At any time in your life, did another child or teen make you do sexual things?

S4) Forced Sex (Including attempts)

At any time in your life, did anyone try to force you to have sex; that is, sexual intercourse of any kind, even if it didn’t happen?

S5) Flashing/Sexual Exposure

At any time in your life, did anyone make you look at their private parts by using force or surprise, or by “flashing” you?

S6) Verbal Sexual Harassment

At any time in your life, did anyone hurt your feelings by saying or writing something sexual about you or your body?

S7) Statutory Rape & Sexual Misconduct (*Note: Suggested for children aged 12 and older.)*

At any time in your life, did you do sexual things with anyone 18 or older, even things you both wanted?

**Exposure to domestic violence (2)**

W1) Witness to Domestic Violence

At any time in your life, did you SEE a parent get pushed, slapped, hit, punched, or beat up by another parent, or their boyfriend or girlfriend?

W2) Witness to Parent Assault of Sibling

At any time in your life, did you SEE a parent hit, beat, kick, or physically hurt your brothers or sisters, not including a spanking on the bottom?

**Additional items added to Enhanced JVQ and used in NATSCEV 1 (Finkelhor 2009): six screeners on EDV**

EF1) Parent Verbally Threatened

At any time in your life, did one of your parents threaten to hurt another parent and it seemed they might really get hurt?

EF2) Parental Displaced Aggression

At any time in your life, did one of your parents, because of an argument, break or ruin anything belonging to another parent, punch the wall, or throw something?

EF3) Parent Pushed

At any time in your life, did one of your parents get pushed by another parent?

EF4) Parent Hit or Slapped

At any time in your life, did one of your parents get hit or slapped by another parent?

EF5) Parent Severely Physically Assaulted

At any time in your life, did one of your parents get kicked, choked, or beat up by another parent?

EF6) Other Family Violence Exposure

Now we want to ask you about fights between any grown-ups and teens, not just between your parents. At any time in your life, did any grown-up or teen who lives with you push, hit, or beat up someone else who lives with you, like a parent, brother, grandparent, or other relative?

**Additional items used in NATSCEV 2 (Finkelhor 2014): four screeners on neglect**

M5) Neglect from Parental Incapacitation

Was there a time in your life that you often had to look after yourself because a parent drank too much alcohol, took drugs, or wouldn't get out of bed?

M6) Neglect from Parental Absence

Was there a time in your life when you often had to go looking for a parent because the parent left you alone, or with brothers and sisters, and you didn't know where the parent was?

M8) Neglect from Unsafe Environment

Was there a time in your life when you lived in a home that was broken down, unsafe, or unhealthy. For example, it had broken stairs, toilets or sinks that didn't work, trash piled up, and things like that?

M9) Neglect from Lack of Hygiene Supervision

Was there a time in your life when your parents did not care if you were clean, wore clean clothes, or brushed your teeth and hair?

**Additional items used in NATSCEV 3 (Finkelhor 2015): one screener item on sexual abuse**

S8) Has anyone ever had sex or tried to have sex with you when you didn’t want it, when you were very high, drunk, or drugged?

**2. Conflict Tactics Scale Parent-Child version (CTSPC) and**

**Conflict Tactics Scale 2 (CTS2)**

Two studies used the Conflict Tactics Scale Parent-Child version to measure SA, PA, E/PA, and Neglect, and the Conflict Tactics Scale 2 to measure EDV: Chan 2011, Chan et al. 2011.

Both **Chan 2011**, and **Chan et al. 2011** adopted the following items from the CTS-PC. We located the CTS-PC from the original article: Straus MA, Hamby SL, Finkelhor D, Moore DW, Runyan D. Identification of Child Maltreatment with the Parent-Child Conflict Tactics Scales: Development and Psychometric Data for a National Sample of American Parents. Child Abuse Negl 1998; 22: 249-270. The relevant items are at Appendices 2 and 3 which contain items on psychological aggression (5 items, p. 268); physical assault by corporal punishment (5 items, p. 268); physical maltreatment (8 items, p. 268); and neglect (5 items, p. 269); and Straus et al. (1996) Appendix Part 3 (pp. 310-312) contains the EDV scale.

For SA: although the CTS-PC does contain SA items, these were not used in the Chan studies.

For PA: 13 items

H. Spanked him/her on the bottom with your bare hand

D. Hit him/her on the bottom with something like a belt, hairbrush, a stick or some other hard

object

P. Slapped him/her on the hand, arm, or leg

R. Pinched him/her

C. Shook him/her (this is scored for Very Severe If the child is <2 years)

V. Slapped him/her on the face or head or ears

0. Hit him/her on some other part of the body besides the bottom with something like a belt,

hairbrush, a stick or some other hard object

T. Threw or knocked him/her down

G. Hit him/her with a fist or kicked him/her hard

K. Beat him/her up, that is you hit him/her over and over as hard as you could

I. Grabbed him/her around the neck and choked him/her

M. Burned or scalded him/her on purpose

S. Threatened him/her with a knife or gun

For E/PA: 5 items

N. Threatened to spank or hit him/her but did not actually do it

F. Shouted, yelled, or screamed at him/her

J. Swore or cursed at him/her

U. Called him/her dumb or lazy or some other name like that

L. Said you would send him/her away or kick him/her out of the house

For neglect: 5 items

NA. Had to leave your child home alone, even when you thought some adult should be with

him/her

NB. Were so caught up with your own problems that you were not able to show or tell your child that you loved him/her

NC. Were not able to make sure your child got the food he/she needed

ND. Were not able to make sure your child got to a doctor or hospital when he/she needed it NE. Were so drunk or high that you had a problem taking care of your child

For EDV:

Both studies by Chan adopted items from the CTS2 for EDV. We located the CTS2 in its published form (the items are in Appendix 3, p. 310): Straus MA, Hamby SL, Boney-McCoy S, Sugarman DB. The Revised Conflict Tactics Scales (CTS2). J Fam Issues 1996; 17: 283-316. The relevant items are at pp. 310-312.

The CTS 2 contains 78 items, asking the same 39 items about actions by each parent. In the Chan studies, the child was asked if she or he had experienced these 39 events by either parent. Accordingly, we treat these as 39 items for the purpose of this study. The Chan studies state that “partner violence was defined as acts of physical assault, psychological aggression, or injury perpetrated by a current marital or cohabiting partner, as measured by the revised Conflict Tactics Scale (CTS2)”. The CTS 2 contains some items that appear directed towards sexual aggression, but could also be interpreted as psychological aggression. The Chan studies do not list the specific items they asked, but nor do they state that items were excluded. Accordingly, for our purposes we assume all 39 items were used. Here we have extracted one item form each of the 39 pairs.

2. My partner showed care for me even though we disagreed.

4. My partner explained his or her side of a disagreement to me.

5. I insulted or swore at my partner.

7. I threw something at my partner that could hurt.

9. I twisted my partner's arm or hair.

12. My partner had a sprain, bruise, or small cut because of a fight with me.

14. My partner showed respect for my feelings about an issue.

15. I made my partner have sex without a condom.

17. I pushed or shoved my partner.

19. I used force (like hitting, holding down, or using a weapon) to make my partner have oral or anal sex.

21. I used a knife or gun on my partner.

23. I passed out from being hit on the head by my partner in a fight.

26. My partner called me fat or ugly.

27. I punched or hit my partner with something that could hurt.

29. I destroyed something belonging to my partner.

31. I went to a doctor because of a fight with my partner.

33. I choked my partner.

35. I shouted or yelled at my partner.

37. I slammed my partner against a wall.

39. I said I was sure we could work out a problem.

42. My partner needed to see a doctor because of a fight with me, but didn't.

43. I beat up my partner.

45. I grabbed my partner.

47. I used force (like hitting, holding down, or using a weapon) to make my partner have sex.

49. I stomped out of the room or house or yard during a disagreement.

51. I insisted on sex when my partner did not want to (but did not use physical force).

53. I slapped my partner.

55. I had a broken bone from a fight with my partner.

57. I used threats to make my partner have oral or anal sex.

59. I suggested a compromise to a disagreement.

61. I burned or scalded my partner on purpose.

63. I insisted my partner have oral or anal sex (but did not use physical force).

65. I accused my partner of being a lousy lover.

67. I did something to spite my partner.

69. I threatened to hit or throw something at my partner.

71. I felt physical pain that still hurt the next day because of a fight with my partner.

73. I kicked my partner.

75. I used threats to make my partner have sex.

77. I agreed to try a solution to a disagreement my partner suggested.

**3. ICAST-CH**

Two studies (Feng 2015; Nikolaidis 2018) used the ICAST (Child version), which we located at Zolotor A, Runyan D, Dunne MP, et al. ISPCAN Child Abuse Screening Tools Children’s Version (ICAST-C): Instrument development and multi-national pilot testing. Child Abuse Negl 2009; 33: 833–841.

**Feng 2015** used the following items; notes in parentheses indicate the modifications used in **Nikolaidis 2018**:

SA: 6 items (Nikolaidis used 5 items for 11 year olds*, and all 6 items for 13 and 16 year olds)

- Talked to you in a sexual way*
- Shown pornography*
- Looked at private parts*
- Touched private parts*
- Made a sex video of you
- Forced sex behaviors*

PA: 9 items (Nikolaidis used 16 items for 11 year olds, and 17 items for 13 and 16 year olds)

- Pushed, grabbed, kicked
- Hit, beat, spanked with hand
- Hit, beat, spanked with object
- Tried to choke, smother, or drown
- Burned or scalded
- Locked in small place
- Pulled hair, pinched, twisted ear
- Held heavy load as punishment
- Threatened with knife or gun

E/PA: 8 items (Nikolaidis used 17 items for 11 year olds, and 19 items for 13 and 16 year olds items*)

- Screaming
- Insulted
- Made you feel embarrassed
- Wished you were dead
- Threatened to abandon
- Locked out of home
- Threatened to hurt or kill you
- Bullied by another child at home

Neglect: 6 items (Nikolaidis used 4 items*)

- Went hungry or thirsty
- Inadequate clothing
- Unmet medical need*
- Felt not cared for*
- Felt unimportant*
- Inadequate support/help*

EDV: 7 items (Nikolaidis used 0 items)

- Frightened by adults’ using drugs
- Adults shouted in frightening way
- Witnessed adults in home hit, kick, slap
- Witnessed adults in home using weapons
- Someone close got killed near home
- Saw people being shot or rioting
- Something stolen from home

**4. Childhood Trauma Questionnaire**

Two studies used the Childhood Trauma Questionnaire (short-form version, located in Bernstein DP, Stein JA, Newcomb MD, et al. Development and validation of a brief screening version of the Childhood Trauma Questionnaire. Child Abuse Negl 2003; 27: 169-190). **Hauser 2011** and **Witt 2017** used the following:

SA: 5 items:

- Was touched sexually
- Hurt if didn’t do something sexual
- Made to do sexual things
- Was molested
- Was sexually abused

PA: 5 items:

- Hit hard enough to see doctor
- Hit hard enough to leave bruises
- Punished with hard objects
- Was physically abused
- Hit badly enough to be noticed

E/PA: 5 items:

- Called names by family
- Parents wished was never born
- Felt hated by family
- Family said hurtful things
- Was emotionally abused

Neglect: 10 items: (reverse scored)

- Felt loved
- Made to feel important
- Was looked out for
- Family felt close
- family was source of strength
- Not enough to eat
- Got taken care of
- Parents were drunk or high
- Wore dirty clothes
- Got taken to doctor

Hauser reports the psychometric data for the translated instrument, and for the original CTQ as reported by Bernstein et al.^67^ Hauser does not further define the constructs, although the German version of the instrument is appended, adapting Bernstein’s CTQ (p. 179-80). Bernstein’s definitions of maltreatment types have shortcomings of vagueness and breadth: p. 175). The CTQ short-form was designed for use with clinical patients rather than whole populations.

**5. Lifestyle and Attitudes Towards Sexual Behavior**

One study used this instrument. **Tsuboi 2015** did not report the items in detail, but described them in the following way, from which we extracted items to the most realistic extent possible (p. 2580).

“Specifically, participants were asked to select from various types of child abuse that they may have experienced when less than 18 years of age. These types of child abuse fell into the following 4 categories: physical abuse, such as being hit or kicked, having boiling water poured on oneself, or experiencing cigarette burns; sexual abuse, such as being forced to conduct sexual acts, witnessing sexual acts, or being forced to look at genitals; neglect, such as not being given food or being left alone for a long period of time; and psychological abuse, such as being reviled repeatedly.”

SA: 1 item:

- being forced to conduct sexual acts, witnessing sexual acts, or being forced to look at genitals

PA: 1 item:

- being hit or kicked, having boiling water poured on oneself, or experiencing cigarette burns

E/PA: 1 item:

- being reviled repeatedly

Neglect: 1 item:

- not being given food or being left alone for a long period of time

Note also that Tsuboi’s study included individuals aged 16-19 in the sampling frame but analysis excluded their data.

**6. Self-developed tools**

Two studies used an instrument they developed independently: Christofferson 2013; May-Chahal 2005.

**Christofferson 2013** used the following:

SA: 4 items (limited to SA by a parent or a guardian):

- Experienced sexual touching or someone exposing their private parts /sex organs to you
- Experienced attempted intercourse
- Experienced forced / completed intercourse
- Experienced other types of sexual behaviour

PA: 7 items:

- Beaten with an object, such as a whip or coat hanger?
- Threatened with a weapon, such as a knife or a gun?
- Had objects thrown at you?
- Grabbed round the neck and chocked?
- Been left with burn or bite marks?
- Had injuries such as broken bones, stab wounds, brain haemorrhage, or burns which were treated by a doctor?
- Been hit, kicked or exposed to violence which has resulted in bruising, bleeding, or other physical injuries?

E/PA: 6 items:

- Addressed in humiliating (e.g. being called lazy, stupid, or useless) manner by parents/guardians
- Humiliated or degraded in public by parents/guardians
- Threatened about getting thrown out of the home by parents/guardians
- Threatened about violent punishment by parents/guardians
- Parents/guardians have through their behaviour shown that you were unwanted, unloved, and worthless
- Parents/guardians have critized or bullied you constantly

Neglect: 7 items:

- Aged <12 you were expected to wash own clothes
- Aged <12 you had to attend school in dirty clothes because there were no clean ones available
- Aged <12 you were occasionally starved due to lack of food or no one available to prepare meals
- Aged <12 you were responsible for own care when sick
- Aged <12 had to call a doctor for yourself when ill
- Often had to care for yourself due to parental alcohol or drug problems
- Were often abandoned in the home for several days

**May-Chahal 2005** used the following:

SA: 14 items:

- Pornographic photos or videos were taken of you
- Shown pornographic images
- Made or encouraged to watch people performing sex acts (not pictures)
- Someone exposed their sex organs or other private parts of their body to you in order to shock you or excite themselves
- You were hugged or kissed in a sexual way
- Someone touched or fondled your sex organs or other private parts of your body
- Someone got you to touch their sex organs or sexually arouse them with your hand
- Someone attempted oral sex on you
- Someone attempted sexual intercourse with you
- Someone attempted anal intercourse with you
- You had full sexual intercourse
- You had anal intercourse
- You had oral sex
- Someone put their finger, tongue or an object into your vagina or anus

PA: 9 items:

- Hit on bottom with hard implement
- Hit on another part of the body with a hard implement
- Shaken
- Hit with fist/kicked hard
- Thrown/knocked down
- Beaten up/hit over and over again
- Grabbed round the neck and choked
- Burned or scalded on purpose
- Threatened with a knife or gun

E/PA: 7 items:

- Psychological control and domination (attempts to control child’s thinking and isolation from sources of support and development)
- Psycho/physical control and domination (physical acts exerting control causing distress rather than physical injury)
- Humiliation/degradation (psychological attacks on self-worth, self-esteem)
- Withdrawal (withholding affection and care, exclusion from the family)
- Antipathy (showing marked dislike of the child by word or deed)
- Terrorizing (threats to harm the child or someone/something the child loves, threats of fear figures, being sent away or making child do something that frightens them)
- Proxy attacks (harming someone/something child values)

Neglect: 8 items:

- Aged <12 always/often went hungry because no one got you meals ready or there was no food in the house
- Aged <12 always/often were ill but no one looked after you or took to doctors
- Aged <12 always/often went to school in dirty clothes because there were no clean ones available
- Often had to look after themselves because parents had problems, for example, alcohol or drugs
- Regularly had to look after self because parents went away
- Allowed to go into dangerous places or situations
- Abandoned or deserted
- Physical condition of their home was dangerous

**7. Blended tools**

Four studies used an instrument that blended aspects of several tools: Denholm 2013; Euser 2015; Shen 2016; Van der Kooij 2015.

**Denholm 2013** used the following:

SA: 1 item:

I was sexually abused by a parent (where “sexual abuse” had been defined as: “Any completed or attempted sexual act, sexual contact or non-contact sexual interaction with a child by a caregiver”

PA: 1 item:

- I was physically abused by a parent – punched, kicked or hit or beaten with an object, or

needed medical treatment

E/PA: 2 items:

- I was verbally abused by a parent
- I suffered humiliation, ridicule, bullying or mental cruelty from a parent

Neglect: 11 items (different items depended on whether the data were collected from the parent and the teacher when the participant was a child, or from the participant directly when aged 45):

Participant:

- I was neglected (where “neglect” was defined as “Failure to meet a child’s basic physical, emotional, medical/dental or education need; failure to provide adequate nutrition, hygiene or shelter; or failure to ensure a child’s safety”)
- Mother unaffectionate
- Father unaffectionate

Parent:

- Mother hardly ever reads to child
- Father hardly ever reads to child
- Hardly ever takes outings with mother
- Hardly ever takes outings with father
- Low parental aspirations

Teacher:

- Child’s appearance is scruffy/dirty/underfed
- Mother little interest in education
- Father little interest in education

EDV: 1 item:

- I witnessed physical or sexual abuse of others in my family

(Denholm used the instrument from the Path Through Life Project; further based on the Parental Bonding Instrument, the British National Survey of Health & Development, and the US National Comorbidity Survey: see p. 342-3. Note that neglect items varied by wave, and by participant (parent, teacher, cohort member); three neglect items were used with the participant at age 45.)

**Euser 2015** compared three sources of CM rates: sentinel reports, CPS reports, and self-reports. Three tools were blended for the self-report component of the study, although it was not reported in the article exactly how this was done. Euser states the questionnaire “was based on the one used in the PoA-2005 study [and] consisted of 24 questions about different types of maltreatment based on the Dating Violence Questionnaire…and the Parent-Child Conflict Tactics Scales…”. The study did not report the number or nature of questions asked about each maltreatment type. However, we contacted the authors of the study and they provided the instrument used (copy on file with authors). We extracted the data from this provided instrument for the purposes of our study.

**Shen 2016** used the following:

SA: 2 items:

- Someone showed me pornographic magazines, pictures, videos, or Internet sites when I did not want to see them.
- Someone touched my private parts or made me touch their private parts when I didn’t want them to.

PA: 7 items:

- Dad (Mom) slapped me on the face, head, or ears (3 times or more).
- Dad (Mom) hit or spanked me with something like a belt, rod, or something hard (3 times or more).
- Dad (Mom) pushed, grabbed, or shoved me to hurt me (3 times or more).
- Dad (Mom) threw something at me to hurt me (3 times or more).
- Dad (Mom) kicked, bit, or punched me to hurt me.
- Dad (Mom) choked, burned, cut, or stabbed me with a knife or something sharp like that.
- Dad (Mom) did something else with the intention and the effect of physically hurting me badly.

E/PA: 4 items:

- Dad (Mom) swore at me or called me names.
- Dad (mom) said that he (she) wished I were dead or had never been born.
- Dad (Mom) threatened to abandon me.
- Dad (Mom) locked me out of our home.

Neglect: 4 items:

- Family members left me home alone.
- I did not get enough to eat.
- I had to wear dirty or torn clothes or clothes that were too warm or not warm enough.
- When I was sick or hurt, I was not taken to see a doctor or not given the medicines I needed.

EDV: 2 items

- I saw or heard my parents calling each other names.
- I saw or heard my parents hitting each other.

**Van der Kooij 2015** used the following:

SA: 7 items:

Within family

- Forced by adult to look at/touch his/her private parts
- Had sex with adult
- Sexual abuse by person under age
- Forced by person under age to look at/touch his/her private parts

Outside family

- Sexual abuse by person under age
- Forced by adult to look at/touch his/her private parts
- Forced by person under age to look at/touch his/her private parts

PA: 8 items: (physical assault by parent)

- Hit with a fist or being kicked hard
- Thrown or knocked down
- Hit at bottom with belt or other hard object
- Beat up (hit the child over and over as hard as they could)?
- Hit on some other part of the body besides the bottom with something like a belt, hairbrush, stick, or some other hard object?
- Grabbed around the neck and choked
- Threatened with knife or gun
- Burned or scalded on purpose by glowing object

E/PA: 1 item: (psychological aggression of parents)

- Parent threatened to spank or hit but did not actually do it

Neglect: 8 items:

- No help of parent with homework
- Not stimulated by performance by parents
- Parents indifferent for problems at school
- Not reassured by grief by parents
- No help of parents when having problems
- Parents did not look after child looking properly
- Parents did not look after personal hygiene of child
- Parents did not look after regular schooling

EDV: 7 items:

- Parent has pushed the other (hard) or had gripped the other
- Parent has beaten the other
- Parent has kicked, bitten or punched the other
- Parent has tried to beaten the other with an object
- Parent has beaten up the other
- Parent has threaten the other with knife or gun
- Parent has used knife or gun to the other

(Van der Kooij used a blend of the instrument used in Euser, the CTSPC, and the Dating Violence Questionnaire).

**8.** **Adverse Childhood Experiences International Questionnaire**

One study used the ACE-IQ (**Al Muneef 2017**). Note that while the ACE-IQ has been used in various forms, data on the psychometric properties of the full version of the ACE-IQ are limited (Meinck et al. 2017).

SA: 4 items

- A5 Did someone touch or fondle you in a sexual way when you did not want them to?
- A6 Did someone make you touch their body in a sexual way when you did not want them to?
- A7 Did someone attempt oral, anal, or vaginal intercourse with you when you did not want them to?
- A8 Did someone actually have oral, anal, or vaginal intercourse with you when you did not want them to?

PA (2 items)

- A3 Did a parent, guardian or other household member spank, slap, kick, punch or beat you up?
- A4 Did a parent, guardian or other household member hit or cut you with an object, such as a stick (or cane), bottle, club, knife, whip etc?

E/PA (2 items)

- A1 Did a parent, guardian or other household member yell, scream or swear at you, insult or humiliate you?
- A2 Did a parent, guardian or other household member threaten to, or actually, abandon you or throw you out of the house?

Neglect (4 items)

- P2 Did your parents/guardians really know what you were doing with your free time when you were not at school or work?
- P3 How often did your parents/guardians not give you enough food even when they could easily have done so?
- P4 Were your parents/guardians too drunk or intoxicated by drugs to take care of you?
- P5 How often did your parents/guardians not send you to school even when it was available?

EDV (3 items)

- F6 Did you see or hear a parent or household member in your home being yelled at, screamed at, sworn at, insulted or humiliated?
- F7 Did you see or hear a parent or household member in your home being slapped, kicked, punched or beaten up?
- F8 Did you see or hear a parent or household member in your home being hit or cut with an object, such as a stick (or cane), bottle, club, knife, whip etc.?

1. **Adverse Childhood Experiences**

One study used the ACE (**Nagy 2019**). Items were introduced by asking whether these experiences occurred during the first 18 years of life.

EA: 1 item

Did a parent or other adult in the household often or very often . . . Swear at you, insult you, put you down, or humiliate you? Or Act in a way that made you afraid that you might be physically hurt?

PA: 1 item

Did a parent or other adult in the household often or very often . . . Push, grab, slap, or throw something at you? Or Ever hit you so hard that you had marks or were injured?

SA: 1 item

Did an adult person at least 5 years older than you ever . . . Touch or fondle you or have you touch their body in a sexual way? Or Attempt or actually have oral, anal, or vaginal intercourse with you?

Neglect: 2 items

Did you often or very often feel that . . .

- You didn’t have enough to eat, had to wear dirty clothes, and had no one to protect you? or Your parents were too drunk or high to take care of you or take you to the doctor if you needed it?
- No one in your family loved you or thought you were important or special? or Your family didn’t look out for each other, feel close to each other, or support each other?

EDV: 1 item

Was your mother or stepmother: Often or very often pushed, grabbed, slapped, or had something thrown at her? Or Sometimes, often, or very often kicked, bitten, hit with a fist, or hit with something hard? Or Ever repeatedly hit for at least a few minutes or threatened with a gun or knife?
