# Supplementary material for: Improving measurement of child abuse and neglect: A systematic review and analysis of national prevalence studies
Source: PLoS One. 2020 Jan 28;15(1):e0227884. doi: 10.1371/journal.pone.0227884 (PMC6986759; doi:10.1371/journal.pone.0227884)
Supplement: S4 File — (DOCX) [file pone.0227884.s005.docx]

**S4 File. Quality assessment tool**

| **Was the study’s target population a close representation of the national population in relation to relevant variables, e.g. age, sex**?   - Nationally representative=2 - Captured estimates for a rural and urban sample =1 - Captures estimates for only a rural or urban sample =0 |
| --- |
| **Was the sampling frame a true or close representation of the target population?**   - Yes=1 - No=0 |
| **Was some form of random selection used to select the sample, OR, was a census undertaken?**   - Yes=1 - No=0 |
| **Was the likelihood of non-response bias minimal?**   - 60% response rate or higher =1* - 59% or lower =0** - Multi-country study with different response rates above and below 60%: 0.5 |
| **Were data collected directly from the subjects (as opposed to a proxy)?**   - Directly =1 - Directly for some subjects, proxy for other subjects=0.5 - Proxy =0 |
| **Were the questions asked about each form of child abuse and neglect in the instrument broadly congruent with generally accepted understandings of these concepts?*****   - Yes=1 - No=0 |
| **Was the study instrument that measured the parameter of interest (e.g. prevalence of child sexual abuse) shown to have reliability and validity (if necessary)?**   - Yes=1 - No=0 |
| **Was the same mode of data collection used for all subjects?**   - Yes=1 - No=0 |
| **Were the numerator(s) and denominator(s) for the parameter of interest appropriate?**   - Yes=1 - No=0 |

*The response rate was >/=60% OR, an analysis was performed that showed no significant difference in relevant demographic characteristics between responders and non-responders. Note that studies conducted in schools did not report the response rate due to difficulty in calculation. Here, we drew on the schools’ participation rate and the students’ participation rate within participating schools; in most cases, this resulted in a rating of 1. One household study did not report the response rate, but informed the authors it was over 70%.

**The response rate was <60%, and if any analysis comparing responders and non-responders was done, it showed a significant difference in relevant demographic characteristics between responders and non-responders.

***Note that we used the following approach for this item, based on the following premises:

1. there are challenges in arriving at an “acceptable” case definition for each type of maltreatment. There are not universally accepted, simple definitions or conceptual models for each type. However, there are some generally acknowledged conceptual understandings or models of each type, that have more sophistication and weight than others, and they are in either in articles in leading international journals or in publications by organisations like the WHO;
2. a well-established principle in the literature, which should inform assessment of this criterion, is that prevalence studies of child maltreatment that only ask one general non-specific item (such as “were you sexually abused”) are not sufficiently behaviourally specific and are likely to produce inaccurate responses which underestimate true prevalence (e.g., Fisher, 2009);
3. some of the included studies covered four types of maltreatment, and some covered five.

Our approach for this criterion asked: Were the questions asked about each form of child abuse and neglect broadly congruent with generally accepted understandings of these concepts?

Therefore:

1. We referred to generally recognised conceptual understandings of each maltreatment type, referring to either published articles or major organizational approaches, which we explained in the article, and used these as the standard against which to measure the specific questions posed for each kind of abuse or neglect.
2. We also considered in this process the “single non-specific question” principle;
3. We agreed that it would be inaccurate and too harsh to give a 0 if the study’s questions are deficient in even one maltreatment type;
4. We agreed that it would be inaccurate and too lenient to give a 1 if the study is deficient in half or more of the maltreatment types it measures;

Ultimately, in arriving at the score for this criterion, we rated a study as 0 if, for three or more kinds of maltreatment, the study either:

- asked only one single non-specific question; OR
- asked a series of questions which *are not* generally congruent with the generally acknowledged conceptual understanding of the maltreatment types.

A study was rated as 1 if, for three or more kinds of maltreatment, the study either:

- asked a single question which nevertheless contained several specific depictions of the type of maltreatment; OR
- asked a series of questions which *are* generally congruent with the generally acknowledged conceptual understanding of the maltreatment types.

Note also that for categories of maltreatment which themselves have multiple distinct sub-categories – e.g. neglect, and emotional abuse – we considered whether the questions asked covered at least half of the subcategories.
