# Supplementary material for: Improving measurement of child abuse and neglect: A systematic review and analysis of national prevalence studies
Source: PLoS One. 2020 Jan 28;15(1):e0227884. doi: 10.1371/journal.pone.0227884 (PMC6986759; doi:10.1371/journal.pone.0227884)
Supplement: S5 File — (DOCX) [file pone.0227884.s006.docx]

**S5 File. Prevalence rates of included studies**

**Notes**

The prevalence rates found by the included studies are presented here as they were detailed in the published articles. We would caution against direct comparison, given the differences in the methodologies and samples. In addition, there are numerous salient differences in the items asked, which will have a profound impact on comparability. Many of these are indicated in the notes to the table below.

However, we recognise readers may be interested in the studies’ findings. In addition, some findings that are either very high or very low, or are outliers, may be consistent with arguments in our manuscript about optimal approaches to measurement. For example, we discouraged approaches to constructs of a maltreatment type that go beyond the parameters of a robust conceptual model (e.g., through the use of vague or overinclusive concepts) which are likely to lead to overestimates of a type of maltreatment. Similarly, we discouraged the use of single items, which are likely to lead to underestimates of a type of maltreatment.

Note also that, for simplicity:

- We have not included rates for those studies that provided only single year data (Euser et al 2013, Finkelhor et al 2005, Shen et al 2016);
- Where studies asked both adults about their childhood experiences, and children of various ages, we used the adult figure (Radford et al 2013, van der Kooij et al 2015);
- We have not included rates for those studies that do not provide complete prevalence rates. Hauser et al 2011 and Witt et al 2017 reported prevalence rates by severity (none-minimal, low-moderate, moderate-severe, and severe-extreme). For reasons that are not fully clear, in reporting prevalence, they counted only those falling within either moderate-severe or severe-extreme categories.
- Where studies report physical and emotional neglect separately and have no single rate for neglect, we present a rate that sums both physical and emotional neglect (e.g., Lev-Wiesel et al 2018, Nagy et al 2019);
- In general we have presented the most all-encompassing prevalence rate provided. For example, where a study separately reported both contact and non-contact sexual abuse, we presented a combined figure or the figure most accurately representing the prevalence rate of sexual abuse (e.g., Nikolaidis et al 2018).
- In some studies, exposure to domestic violence includes only physical violence; other studies include verbal violence. If there is a figure that includes both, we have used that figure;
- Where corporal punishment can be separated from physical abuse, we have presented the rate for physical abuse without corporal punishment (Chan et al 2011);
- Exposure to domestic violence is the most inconsistently defined maltreatment type; in some instances the reported rate includes exposure to abuse of siblings. Where the figure appears to include exposure to community violence, we have excluded that part of the rate;
- For full details and context, the article should be consulted.

| Study | Nation | EA (%) | SA (%) | PA (%) | Neglect (%) | EDV (%) |
| --- | --- | --- | --- | --- | --- | --- |
| Almuneef 2017 | Saudi Arabia | 52 | 21 | 42 | 29 | 57^[[1]](#footnote-1)^ |
| Chan 2011 | Hong Kong | 70.9^[[2]](#footnote-2)^ | - | 27.0^[[3]](#footnote-3)^ | 32.3 | 73.2^[[4]](#footnote-4)^ |
| Chan et al 2011 | Hong Kong | 72.0^[[5]](#footnote-5)^ | - | 29.3^[[6]](#footnote-6)^ | 36.0 | 71.6^[[7]](#footnote-7)^ |
| Christoffersen et al 2013 | Denmark | 5.2^[[8]](#footnote-8)^ | 3.4^[[9]](#footnote-9)^ | 5.4^[[10]](#footnote-10)^ | 3.0^[[11]](#footnote-11)^ | - |
| Denholm et al 2013^[[12]](#footnote-12)^ | UK | 12.5 | 2.9 | 9.0 | 4.4^[[13]](#footnote-13)^ | 8.5^[[14]](#footnote-14)^ |
| Feng et al 2015 | Taiwan | 69.2 | 19.8 | 61.4 | 54.6 | 82.2^[[15]](#footnote-15)^ |
| Finkelhor et al 2009 | USA | 11.9 | 9.8^[[16]](#footnote-16)^ | 9.1 | 3.6 | 20.3^[[17]](#footnote-17)^ |
| Finkelhor et al 2014 | USA | 10.3 | 9.5^[[18]](#footnote-18)^ | 8.9^[[19]](#footnote-19)^ | 11.6 | - |
| Finkelhor et al 2015 | USA | 14.5 | 8.4^[[20]](#footnote-20)^ | 9.8 | 11.8 | 19.5^[[21]](#footnote-21)^ |
| Lev-Wiesel et al 2018 | Israel | 31.1 | 18.7 | 18.0 | 34.0^[[22]](#footnote-22)^ | 9.8 |
| May-Chahal et al 2015 | UK | 6^[[23]](#footnote-23)^ | 19^[[24]](#footnote-24)^ | 7^[[25]](#footnote-25)^ | >33^[[26]](#footnote-26)^ | - |
| Nagy et al 2019 | Hungary | 5 | 1 | 5 | 7^[[27]](#footnote-27)^ | 5^[[28]](#footnote-28)^ |
| Nikolaidis et al 2018 | Albania | 68.62 | 11.11 | 59.44 | 25.73 | - |
|  | Bosnia and Herzegovina | 72.51 | 18.68 | 67.68 | 39.63 | - |
|  | Bulgaria | 69.51 | 8.58 | 62.21 | 23.68 | - |
|  | Croatia | 73.04 | 10.18 | 66.73 | 35.30 | - |
|  | Former Yugoslavian Republic of Macedonia (FYROM) | 64.58 | 7.60 | 50.66 | 27.47 | - |
|  | Greece | 83.16 | 15.86 | 76.37 | 37.20 | - |
|  | Romania | 76.67 | 7.90 | 66.94 | 22.59 | - |
|  | Serbia | 68.44 | 8.49 | 69.18 | 28.83 | - |
|  | Turkey | 70.58 | - | 58.38 | 42.62 | - |
| Radford et al 2013^[[29]](#footnote-29)^ | UK | 6.9 | 24.1^[[30]](#footnote-30)^ | 8.4^[[31]](#footnote-31)^ | 16 | 23.7 |
| Schick et al 2016 | Switzerland | 26.5 | 2.8^[[32]](#footnote-32)^ | 22.3 | 6.4 | - |
| Tsuboi 2015 | Japan | 4 | 0.6 | 3 | 0.8 | - |
| van der Kooij et al 2015^[[33]](#footnote-33)^ | Suriname | 61.9 | 27.6^[[34]](#footnote-34)^ | 63.2 | 60.7 | 41.0 |
| Ward et al 2018 | South Africa | 12.56 | 12.04 | 18.04 | 12.18 | 24.58^[[35]](#footnote-35)^ |

1. Witnessing domestic violence against any household member. [↑](#footnote-ref-1)
2. See Chan 2011 (Table 3): this is reported as psychological aggression such as shouting, yelling, cursing and naming. [↑](#footnote-ref-2)
3. Includes physical maltreatment and serious physical maltreatment. Prevalence of corporal punishment, physical maltreatment, and severe physical maltreatment is 44.4%. [↑](#footnote-ref-3)
4. This arguably represents the best measure. Chan 2011 (Table 2) reports multiple separate categories of this without a sum total. These are: physical assault by either parent: 26.0; injury by either parent: 13.1; psychological aggression by either parent: 73.2; physical assault/injury by either parent: 27.4. [↑](#footnote-ref-4)
5. Psychological aggression such as shouting, yelling, cursing and naming. [↑](#footnote-ref-5)
6. Includes physical maltreatment and serious physical maltreatment. Prevalence of corporal punishment, physical maltreatment, and severe physical maltreatment is 45.1%. [↑](#footnote-ref-6)
7. This arguably represents the best measure, and was reported as the rate in text (Chan et al 2011, p. 8. Chan et al 2011 (Table 1) reports multiple separate categories of this without a sum total. These are: physical assault by either parent: 27.1; injury to either parent: 14.0; psychological aggression by either parent: 71.6; physical assault/injury by either parent: 28.4. [↑](#footnote-ref-7)
8. Note that this involved emotional abuse experienced under the age of 12. The reported total figure of 5.2% reflected those who endorsed at least *three items*; no rationale was provided for the three item threshold. Note that the highest single reported figure was 13.6% (threatened about getting thrown out of the home), which arguably represents the best measure. [↑](#footnote-ref-8)
9. Occurring under the age of 24. The reported total figure reflected those who endorsed at least one item. [↑](#footnote-ref-9)
10. Experienced under the age of 12. The reported total figure reflected those who endorsed at least one item. [↑](#footnote-ref-10)
11. Note that this involved neglect experienced under the age of 12. The reported total figure of 3.0% reflected those who endorsed at least *two items*; no rationale was provided for the two item threshold. Note that the highest single reported figure was 5.9% (responsible for own care when sick), which arguably represents the best measure. [↑](#footnote-ref-11)
12. These figures are from the imputation sample: see Denholm et al 2013, p. 344. [↑](#footnote-ref-12)
13. Here, we have listed the physical neglect result only assessed at 45 years (4.4%). Note however that Denholm et al 2013 adopted a complex approach to neglect and it is difficult to isolate a single figure. Physical neglect was reported as 7.6% at 7 and 11 years. Emotional neglect was reported by multiple individual items concerning acts and omissions, some of which are arguably not commonly regarded as neglect, or which are vague (e.g., ‘low parental aspirations’): see p 346. The rates for these various kinds of emotional neglect ranged from 6.5% (hardly ever takes outings with mother, at 7, 11 years) – 34.7% (father little interest in education at 7, 11, 16 years). [↑](#footnote-ref-13)
14. Witnessed abuse of family members. [↑](#footnote-ref-14)
15. Reported as an overall rate for ‘violence exposure’, which included: frightened by adults using drugs (26.4); adults shouted in a frightening way (68.3); witnessed adults in home hit, kick, slap (23.0); witnessed adults in home using weapons (10.6); someone close got killed near home (12.3); saw people being shot or rioting (14.9); something stolen from home (38.5). [↑](#footnote-ref-15)
16. This is the figure for ‘any sexual victimization’ (see Table 2), which includes a range of different experiences by different persons (adults, strangers, peers). Sexual assaults by a known adult were reported separately as 1.2% (Table 3). [↑](#footnote-ref-16)
17. This is the figure for ‘Any witnessed family assault’. [↑](#footnote-ref-17)
18. It is important to note that here we have cited the reported prevalence rate from this study of ‘any sexual victimization’ (9.5%), which is detailed in an accompanying article: Finkelhor, D., Turner, H. A., Shattuck, A. M., & Hamby, S. L. (2013). Violence, crime, and abuse exposure in a national sample of children and youth: An update. JAMA Pediatrics, 167, 614–621 (Table 2). The figure cited in Finkelhor et al 2015 for the sexual abuse aggregate (0.7%) only included JVQ screeners regarding ‘sexual abuse by caregiver’. The 2015 article was focused on reporting rates of child maltreatment by parents and adult caregivers, and excluded victimizations by others including peers, to make the definition more consistent with child protection agency definitions. Accordingly, as acknowledged by the article (p. 1423), the prevalence rate for sexual abuse is lower than normally reported. In this article, the sexual abuse figure covered affirmative response to any of four screeners, related to acts by such parents or caregivers: sexual assault by known adult (S1), nonspecific sexual assault (S2), sexual assault by peer (S3), and rape (attempted or completed; S4). The low prevalence rate (especially compared with the results from Finkelhor et al 2009 and Finkelhor et al 2015) is explained by this qualification. [↑](#footnote-ref-18)
19. Note that the reported physical abuse prevalence rate reflected a broad composite figure of ‘physical abuse by caregiver’ which included positive endorsements of any of the following questions, several of which involved peer assaults: Physical Abuse (including hit, beat, kick, or physically hurt, but excluding spanking; M1, see Appendix A), assault with weapon (C4),assault without weapon (C5), attempted assault (C6), bias attack (C9), peer or sibling assault (P2; this could include an older youth caregiver), nonsexual genital assault (P3), assault by adult (A1), or assault with injury (A2). The prevalence rate for the M1 screener – physical abuse by a parent or caregiver – was not separately reported. [↑](#footnote-ref-19)
20. In this article, sexual abuse is not specifically included as a form of child maltreatment (p. 751). The reported prevalence rate of ‘sexual abuse’ in Table 3 is 0.2%, with a note that cell sizes were too small for analysis. However, the reported prevalence rate of ‘any sexual offense’, which is then detailed to suggest items corresponding with the JVQ, is 8.4% (Table 2). [↑](#footnote-ref-20)
21. This is the figure for ‘Any witnessed family assault’ (Table 5). [↑](#footnote-ref-21)
22. This is the sum of physical neglect (17%) and emotional neglect (17%). [↑](#footnote-ref-22)
23. Emotional maltreatment – scoring on at least four of seven dimensions. [↑](#footnote-ref-23)
24. Includes contact/non-contact sexual abuse and ‘consensual’ sexual behaviour aged 13–15 years with someone 5 or more years older. [↑](#footnote-ref-24)
25. Seriously abused by parent/carer; cf 25% experience of violent treatment from anyone. [↑](#footnote-ref-25)
26. This was reported by the authors as the total prevalence on the basis of experiencing at least one of seven dimensions of emotional maltreatment; they also found that 6% reported experiencing four or more types. [↑](#footnote-ref-26)
27. Sum of emotional neglect and physical neglect. [↑](#footnote-ref-27)
28. This is described as ‘household physical violence’. [↑](#footnote-ref-28)
29. Lifetime prevalence rates were given for age under 11, age 11–17, and 18–24. These figures are for those aged 18–24. [↑](#footnote-ref-29)
30. This is the prevalence rate for sexual victimisation by any adult/peer perpetrator; contrast 12.5% contact sexual abuse by any adult/peer; 0.6% sexual abuse by parent/guardian. [↑](#footnote-ref-30)
31. Physical violence from parent/guardian. [↑](#footnote-ref-31)
32. Sexual assault by known adult. [↑](#footnote-ref-32)
33. Lifetime prevalence rates were given separately for adolescents and young adults up to age 22. These figures are for young adults. [↑](#footnote-ref-33)
34. Within family: 20.1; outside family: 21.3. [↑](#footnote-ref-34)
35. Includes violence between parents and between parents and siblings. [↑](#footnote-ref-35)
